# PIF4‐dependent 2‐hydroxymelatonin and 3‐hydroxymelatonin biosynthesis is involved in skotomorphogenic seedling growth in *Arabidopsis thaliana*

**DOI:** 10.1111/tpj.70394

**Published:** 2025-08-01

**Authors:** Hyoung Yool Lee, Kyoungwhan Back

**Affiliations:** ^1^ Department of Molecular Biotechnology, College of Agriculture and Life Sciences Chonnam National University Gwangju 61186 Republic of Korea

**Keywords:** 2‐hydroxymelatonin, 3‐hydroxymelatonin, Arabidopsis, brassinosteroid, giberellin, melatonin, phytochrome interacting factor 4, skotomorphogenesis

## Abstract

Plants enzymatically synthesize melatonin and its hydroxylated forms, such as 2‐hydroxymelatonin (2‐OHM) and 3‐hydroxymelatonin (3‐OHM). Interestingly, 2‐OHM and 3‐OHM are present in plants at higher concentrations than melatonin and are induced at night, which is consistent with the nocturnal induction of melatonin in animals. However, the biological functions of 2‐OHM and 3‐OHM in plants are unclear. Here, we cloned the *melatonin 2‐hydroxylase* (*M2H*) gene responsible for 2‐OHM synthesis in Arabidopsis and generated a double knockout mutant (*m2hm3h*) lacking *M2H* and *melatonin 3‐hydroxylase* (*M3H*), which is responsible for 3‐OHM synthesis. *M2H* and *M3H* were expressed at maximum levels at night, but their expression was greatly suppressed in the *pif4pif5* and *bri1*, whereas they were constitutively overexpressed in *phyB*. Therefore, *M2H* and *M3H* expression act in a PIF4/5‐dependent manner. *m2hm3h* had a short hypocotyl phenotype similar to the *pif4pif5* and showed simultaneous decreases in the expression of various growth‐related genes, including *EXP1*. PIF4 bound to the E‐box element of the *M2H* and *M3H* promoters. In addition, *m2hm3h* showed lower *PIF4* transcript and protein levels compared to the wild type, which may be related to the reduced expression of GA‐ and BR‐biosynthetic genes at the seed imbibition stage. To determine the direct effects of 2‐OHM/3‐OHM on GA and BR biosynthesis genes, seeds were imbibed with a mixture of 2‐OHM and 3‐OHM in darkness. The expression of GA and BR biosynthesis genes was significantly induced during the seed imbibition period compared to the control. By discovering PIF4‐dependent *M2H* and *M3H* expression, we elucidate the mechanism by which 2‐OHM and 3‐OHM act as signaling molecules for skotomorphogenic seedling growth in plants.

## Significance Statement

Melatonin 2‐hydroxylase and melatonin 3‐hydroxylase, which are responsible for the synthesis of 2‐hydroxymelatonin and 3‐hydroxymelatonin, are induced during the night under the control of phytochrome interacting factor 4, and both 2‐hydroxymelatonin and 3‐hydroxymelatonin are involved in skotomorphogenic growth in Arabidopsis by upregulating gibberellin and brassinosteroid synthesis genes.

## INTRODUCTION

Melatonin is present in all living organisms tested to date, but its biological functions vary among organisms (Hardeland, 2016; Zhao, Chen, et al., 2019; Zhao, Yu, et al., 2019). In animals, it acts as a chronobiological hormone, whereas plants use it as a biostimulator to orchestrate myriad processes from growth to defense against abiotic and biotic stresses (Mukherjee et al., 2024; Reiter et al., 2024). Its key biosynthetic genes, including *serotonin N‐acetyltransferase* (*SNAT*), have been cloned from diverse species, including animals, plants, bacteria, and archaea (Voisin et al., 1984; Lee *et al*., 2021, 2022; Chen et al., 2023).

For melatonin biosynthesis, both plants and animals use tryptophan as a substrate and require four enzymatic reactions (Zhao, Chen, et al., 2019; Zhao, Yu, et al., 2019). In plants, the first reaction is the synthesis of tryptamine by tryptophan decarboxylase (TDC), followed by serotonin synthesis by tryptamine 5‐hydroxylase (T5H). There are two pathways from serotonin to melatonin: serotonin to *N*‐acetylserotonin (NAS) to melatonin and serotonin to 5‐methoxytryptamine (5‐MT) to melatonin. SNAT uses serotonin and 5‐MT as substrates to catalyze the production of NAS and melatonin, respectively. NAS *O*‐methyltransferase (ASMT) catalyzes the transformation of serotonin and NAS into 5‐MT and melatonin, respectively. Due to the low catalytic activity of SNAT and ASMT relative to TDC and T5H, the synthesis of melatonin is lower than that of serotonin. For example, the melatonin level in rice is as low as 0.3 ng per gram fresh weight (FW), compared to about 600 ng/g FW serotonin in unstressed seedlings (Back, 2021). The melatonin level is markedly lower in *Arabidopsis thaliana* (hereafter Arabidopsis) and wheat, at 0.025 ng/g FW (Yao et al., 2021) and 0.05 ng/g FW (Li, Brestic, et al., 2018), respectively. Such low levels are comparable to those in the plasma of animals (0.03–0.07 ng/ml) during the night (Kennaway, 2020). The plasma levels of melatonin peak at night in animals but during the day in plants (Lee et al., 2017; Li et al., 2020), indicating that the regulatory mechanisms and biological roles of melatonin biosynthesis differ between plants and animals.

In plants, melatonin is enzymatically metabolized into 2‐hydroxymelatonin (2‐OHM) or 3‐hydroxymelatonin (3‐OHM, also called cyclic 3‐hydroxymelatonin) (Liu et al., 2022). Their levels are at least two orders of magnitude higher than that of melatonin in plants (Byeon, Tan, et al., 2015). Furthermore, when exogenously applied to rice seedlings, melatonin is rapidly metabolized into 3‐OHM and 2‐OHM (Lee et al., 2016). Therefore, treatment of plants with exogenous melatonin leads to the production of multiple melatonin metabolites plus melatonin, which hampers identification of the mode of action of melatonin. For example, in previous studies, exogenous melatonin increased the germination rate in cotton (Xiao et al., 2019) but not in Arabidopsis under unstressed conditions (Lv et al., 2021). However, it enhances germination in a number of plant species under stress, indicating that its function in seed germination depends on the plant species and environmental conditions (Wang et al., 2024). 2‐OHM rather than melatonin increases the germination rate in Arabidopsis by inducing the production of gibberellin and reactive oxygen species (Lee & Back, 2022a). In addition, it confers tolerance against abiotic stresses such as cadmium (Shah et al., 2020) and salt (Korkmaz et al., 2023). The treatment of Arabidopsis with exogenous 3‐OHM transcriptionally induces *FLOWERING LOCUS T* (*FT*) expression, implicating a role in Arabidopsis flowering (Lee & Back, 2022b). These data indicate that melatonin metabolites such as 2‐OHM and 3‐OHM have functions that are distinct from melatonin.

The genes responsible for 2‐OHM and 3‐OHM biosynthesis, such as *melatonin 2‐hydroxylase* (*M2H*) and *melatonin 3‐hydroxylase* (*M3H*) were cloned from rice (Byeon & Back, 2015; Lee et al., 2016) followed by Arabidopsis (Lee & Back, 2022b) and tomato (Yu et al., 2022). Rice *M2H* and *M3H* mRNAs exhibit a dark‐induced diurnal rhythm (Choi & Back, 2019a, 2019b), similar to *SNAT2* in rice (Hwang & Back, 2018). *M2H* overexpression in rice leads to the death of somatic embryogenesis, suggesting that 2‐OHM has a toxic effect (Choi & Back, 2019a). By contrast, overexpression in tobacco results in an accelerated leaf senescence phenotype and enhanced ROS production (Lee & Back, 2021). *M3H* overexpression or downregulation in rice results in phenotypic defects at the reproductive stage, suggestive of the involvement of 3‐OHM in growth and development (Choi & Back, 2019b). Indeed, an Arabidopsis *m3h* exhibits delayed flowering and smaller height than wild type (Lee & Back, 2022b). *M3H* overexpression in tomato leads to an enhanced senescence phenotype by decreasing the melatonin level (Yu et al., 2022). These data suggest that 2‐OHM and 3‐OHM are not merely waste products of melatonin but have distinct functions in growth and development in plants.

Plant growth is regulated by the light/dark cycle and hormonal signals (Xu et al., 2015). In darkness, skotomorphogenic development occurs by inducing two classes of photomorphogenesis repressors—the CONSTITUTIVELY PHOTOMORPHOGENIC 1 (COP1) and PHYTOCHROME INTERACTING FACTOR (PIF) proteins. COP1 and PIF have key functions in hypocotyl elongation in darkness. A series of PIF proteins function as transcription factors by binding promoter *cis*‐sequences (G‐box and E‐box) of target genes (such as auxin biosynthesis genes) and thereby promote seedling growth. By contrast, photomorphogenesis development upon light exposure resumes to inhibit hypocotyl elongation in conjunction with the induction of chlorophyll synthesis for autotrophic growth. Photomorphogenesis begins with the activation of phytochrome B (PhyB) *via* conversion from a red‐absorbing (Pr) to a far‐red‐absorbing (Pfr) form followed by the inhibition of COP1 and PIFs. COP1, a Ring‐type E3 ubiquitin ligase, is a key factor for maintaining the stability of PIF proteins in darkness (Zheng et al., 2016).

The relationship between melatonin and plant growth has been investigated in several plant species (Arnao & Hernández‐Ruiz, 2014). It was hypothesized that melatonin is involved in COP1 regulation in plants and animals because melatonin controls the ubiquitin proteasome system, which regulates thyroid hormone deiodinase, thermogenesis in brown fat, and tumor suppression (Sanchez‐Barcelo et al., 2016). Melatonin induces COP1 under UV‐B stress to protect Arabidopsis (Yao et al., 2021). However, there is no report of a direct relationship between COP1 expression and melatonin in plant growth and development under non‐stress conditions. The direct evidence of the function of melatonin in skotomorphogenesis was from transgenic rice plants in which *SNAT2* was downregulated (Hwang & Back, 2018). The *SNAT2* RNAi rice produced a lower level of melatonin than wild type and exhibited a semi‐dwarf phenotype resulting from a decreased level of brassinosteroid (BR), a key hormone in skotomorphogenic development. However, *SNAT1* RNAi rice did not show skotomorphogenesis despite its decreased level of melatonin compared to wild type. Therefore, melatonin is not a key trigger of skotomorphogenesis (Hwang & Back, 2019).

In this study, we used Arabidopsis for molecular and genetic analyses to investigate the physiological functions of 2‐OHM and 3‐OHM. To this end, we assessed the enzymatic kinetics of Arabidopsis M2H and generated a homozygous *m2hm3h*, which had a shorter hypocotyl phenotype than wild‐type Col‐0 (WT). Because *M2H* and *M3H* transcript levels were low in the *pif4pif5* but high in the *phyB*, we hypothesized that *m2hm3h*‐mediated growth suppression was related to *PIF4*‐dependent skotomorphogenic development. As expected, the phenotype of *m2hm3h* was similar to *pif4pif5*, which had a shorter hypocotyl. Furthermore, the PIF4 transcription factor bound to E‐box elements in the *M2H* and *M3H* promoters, indicating that it was a direct target of PIF4. The findings revealed that the hydroxylated forms of melatonin, such as 2‐OHM and 3‐OHM, are involved in skotomorphogenic growth.

## RESULTS

### *In vitro* enzyme assay and subcellular localization of AtM2H

**Figure 1:**
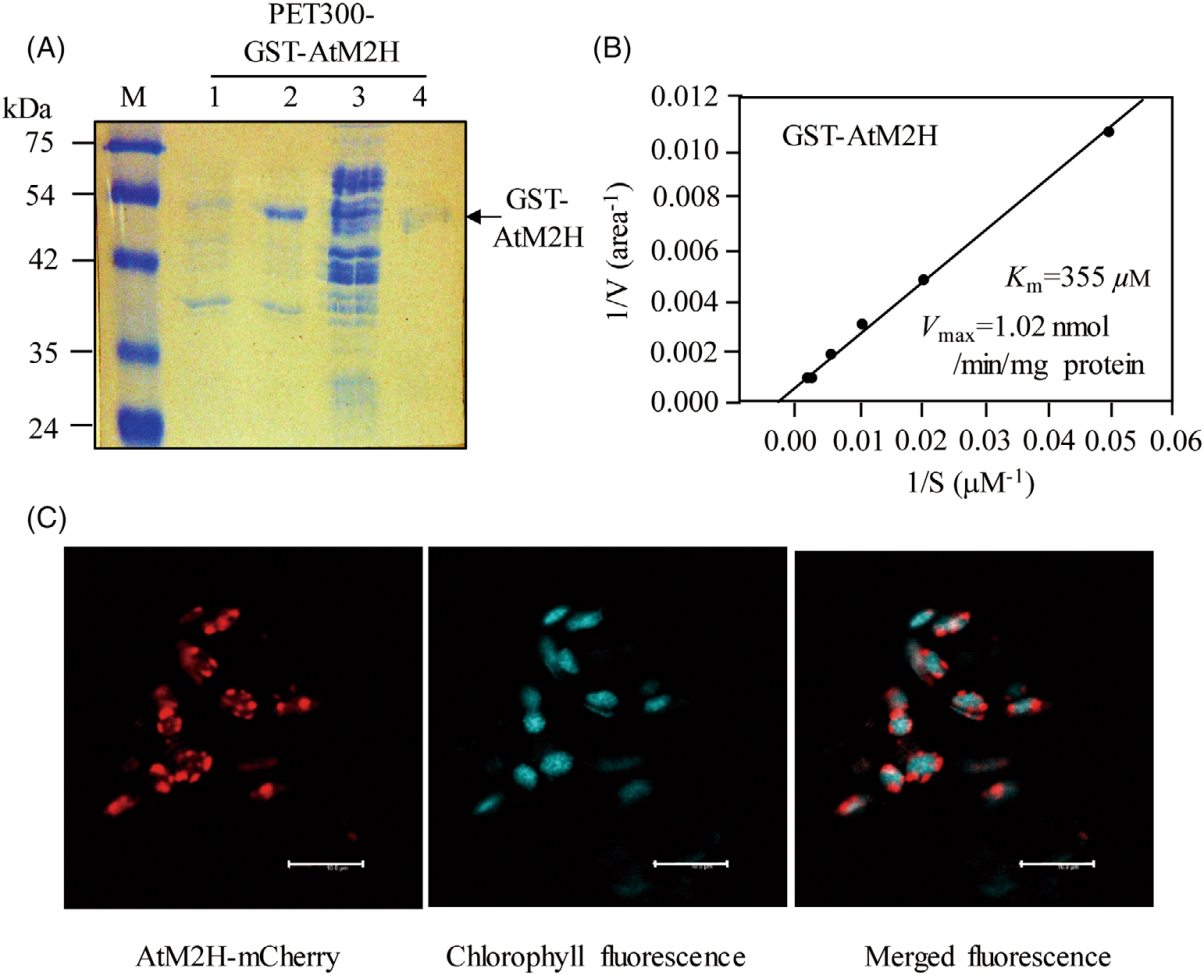
Functional characterization of Arabidopsis *M2H* (AtM2H) candidate gene. (A) Affinity purification of His‐tagged Arabidopsis GST‐AtM2H fusion protein from *E. coli*. Proteins were separated by SDS‐PAGE followed by Coomassie blue staining. M, molecular size marker; lane 1, total *E. coli* protein of uninduced cells (8 × 10^6^ cells) before IPTG induction; lane 2, total *E. coli* protein of IPTG induced cells (8 × 10^6^ cells); lane 3, 20 μg of total soluble protein; lane 4, GST‐AtM2H protein (2 μg) purified by affinity (Ni^2+^) chromatography. An arrow indicates the GST‐AtM2H protein. (B) Kinetics of purified GST‐AtM2H on Lineweaver–Burk plots. (C) Subcellular localization of AtM2H‐mCherry. Left, red fluorescence of AtM2H‐mCherry; middle, cyan fluorescence of chlorophyll; right, merged fluorescence images (left + middle). Tobacco leaves were infiltrated with *Agrobacterium tumefaciens* (GV2260) containing XVE‐inducible AtM2H‐mCherry. Bars, 10 μm.

Arabidopsis M2H (AtM2H; AT3g60290) was selected as a rice M2H homolog by BLAST searching (Altschul et al., 1997) and showed 43% amino acid identity with rice M2H (AK119413) (Byeon & Back, 2015). To determine whether *AtM2H* has M2H activity, *AtM2H* was expressed in *Escherichia coli*, and recombinant M2H protein was purified as a GST‐AtM2H fusion protein by affinity (Ni^2+^) chromatography because expression without the GST tag resulted in insoluble expression (Figure 1A). Purified recombinant GST‐AtM2H showed M2H enzyme activity with *K*_m_ and *V*_max_ values of 355 μm and 1.02 nmol/min mg protein, respectively (Figure 1B). These kinetic parameters were similar to rice M2H (*K*_m_ 145 μm and *V*_max_ 1.11 nmol/min mg protein) (Byeon & Back, 2015). Similar to rice M2H (Byeon, Lee, et al., 2015), AtM2H was localized to the chloroplast (Figure 1C). Therefore, the *AtM2H* gene, a homolog of rice *M2H*, is the bona fide *M2H* gene of Arabidopsis.

### Expression patterns of *M2H* and *M3H* in seedling‐morphogenesis mutants

**Figure 2:**
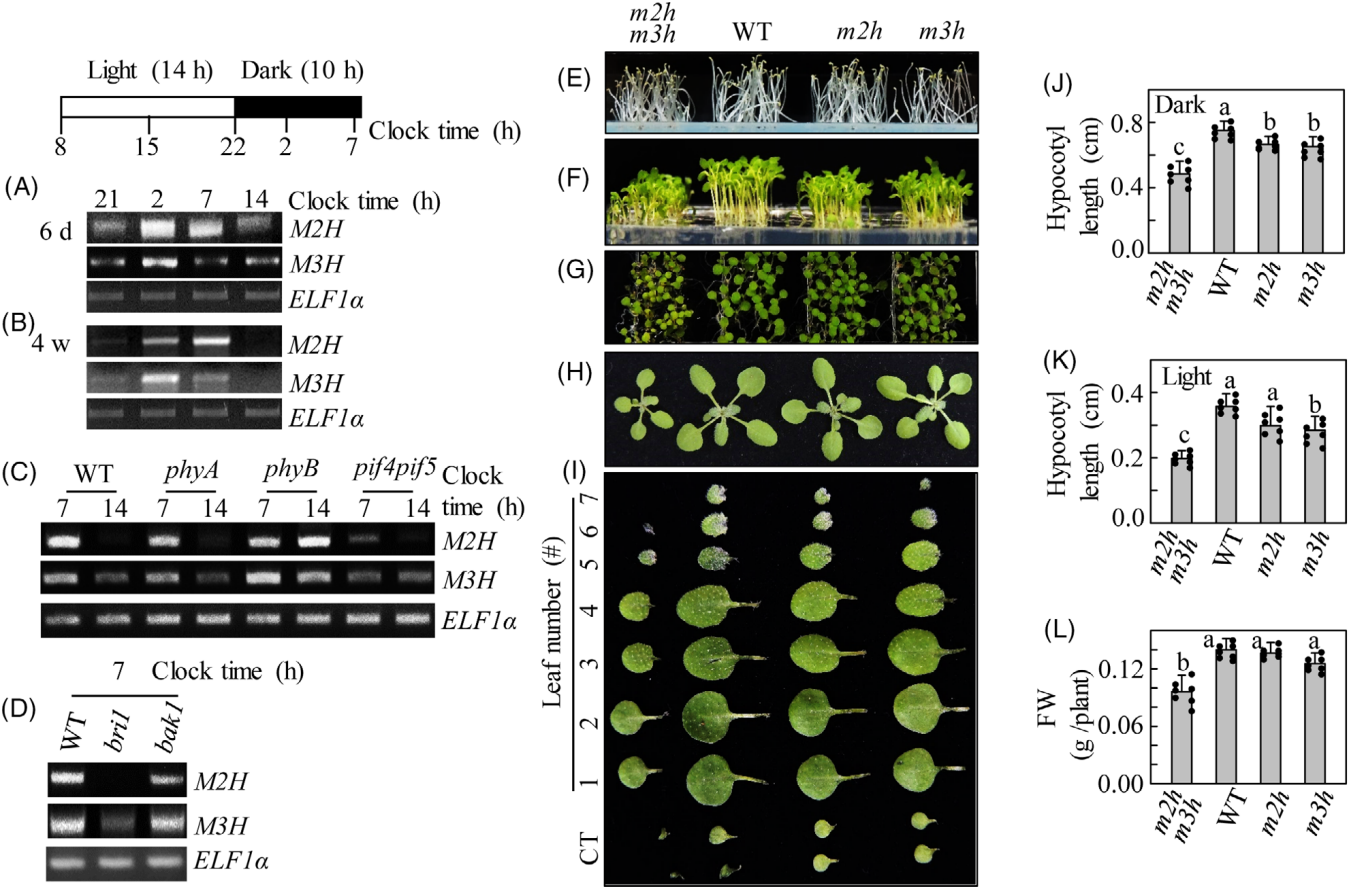
Daily rhythm of *M2H* and *M3H* transcripts and comparison of Arabidopsis seedlings of wild‐type (WT), *m2hm3h*, *m2h* and *m3h* plants grown either in darkness or in light condition. (A, B) Daily rhythm of *M2H* and *M3H* transcript accumulation in WT Arabidopsis plants. (C) Daily rhythm of *M2H* and *M3H* transcript accumulation in *phyA*, *phyB*, and *pif4pif5*. (D) Expression levels of *M2H* and *M3H* transcripts in *bri1* and *bak1*. Transcript levels were measured *via* RT‐PCR assays with *ELF1α* serving as a control. Six‐day‐old seedlings (A) and 4‐week‐old plants (B–D) were harvested at noted time points during day and night periods for RNA extraction, followed by RT‐PCR analysis. (E–I) The seedling phenotypes of WT, *m2h*, *m3h*, and *m2hm3h* on horizontal MS plates grown in darkness for 4 days (E) or under a 14‐h light/10‐dark cycle for 7 days (E, F for side view) and (G, H for top view). Representative Arabidopsis plant phenotypes grown for 4 weeks in soil under a 14‐h light/10‐h dark cycle and leaf shape photos showing leaf size, number of leaves, and petiole length between WT, *m2h*, *m3h*, and *m2hm3h* plants (I). Leaves are arranged in order of leaf emergence from cotyledons (labeled CT) to true leaves (leaf number #1 to #7). Quantification of hypocotyl length of seedlings grown in darkness for 4 days (J) and in light/dark condition for 7 days (K) which collected from samples of (E, F), respectively. Comparison of fresh weight (FW; shoot biomass) (L) of 4‐week‐old plants which collected from samples of (H). Error bars represent mean values (*n* = 7) ± SD and individual datapoints are shown as dots. Different letters indicate significant differences (*p* < 0.05; ANOVA, followed by Tukey's HSD post hoc tests). The white and black boxes in (A) above represent light and dark times, respectively. The numbers below the boxes represent the clock time.

Rice *M2H* and *M3H* expression exhibited diurnal rhythms with nocturnal peaks (Choi & Back, 2019a, 2019b). Thus, we performed reverse transcription polymerase chain reaction (RT‐PCR) analysis to determine whether the expression of *M2H* and *M3H* genes in Arabidopsis also exhibited a night peak. The *M2H* and *M3H* transcript levels were increased at night in 6‐day‐old seedlings. Notably, the diurnal expression pattern with a peak at night was more evident in 4‐week‐old plants than in 6‐day‐old seedlings (Figure 2A). These data suggest that *M2H* and *M3H* have a diurnal induction pattern with a nocturnal peak, suggesting an inhibitory role of photoreceptors. To confirm this idea, the *M2H* and *M3H* transcript levels were investigated in phytochrome mutants. *M2H* and *M3H* were expressed at high levels during the day in the *phyB*, indicating that the diurnal repression of *M2H* and *M3H* transcripts in the WT is regulated by PhyB (Figure 2C). In contrast, the induction of *M2H* and *M3H* expression during the day was not observed in *phyA*. The significant upregulation of *M2H*/*M3H* in the *phyB* is a result of the inhibitory effect of PhyB on *M2H*/*M3H* expression.

PIFs such as PIF4 and PIF5, which are positive transcription factors for hypocotyl growth in darkness, are degraded under light *via* the active phytochrome (Pfr). Accordingly, the nighttime expression of *M2H* and *M3H* was downregulated in the *pif4pif5*, suggesting that *M2H* and *M3H* expression is associated with the PIF4 and PIF5 transcription factors, and that *M2H* and *M3H* may act downstream of PIF4/5 (Figure 2C). Therefore, the repression of *M2H* and *M3H* in the *pif4pif5* is consistent with the increased expression of *M2H* and *M3H* in the *phyB*, as PIF4 acts as a negative regulator of PhyB signaling (Huq & Quail, 2002).

Brassinosteroid (BR) is a hormone that regulates hypocotyl growth in the dark. BR binding to brassinosteroid receptor 1 (BRI1) triggers interaction with BRI1‐associated receptor kinase 1 (BAK1). The BR‐BRI1‐BAK1 complex activates the brassinazole resistance transcription factor (BZR1) and increases PIF4/PIF5 levels, playing a key role in the BR signaling pathway, induction of a large number of target genes, and hypocotyl growth in the dark. In *bri1*, *M2H* expression was not observed, and *M3H* expression was significantly suppressed. In addition, both *M2H* and *M3H* expression were partially suppressed in *bak1* (Figure 2D).

Collectively, the data suggest that the expressions of *M2H* and *M3H* are positively regulated by key regulators of skotomorphogenic development, such as BR and PIF4/5, and negatively regulated by regulators of photomorphogenic development, such as PhyB.

### Growth characteristics of the *m2h*, *m3h*, and *m2hm3h*

To investigate the functional role of *M2H* and *M3H*, we evaluated the phenotypic characteristics of Arabidopsis single mutants (*m2h* and *m3h*) and a double mutant (*m2hm3h*). These mutants were grown with WT plants under constant darkness (4 days) or a 14‐h light/10‐h dark cycle (7 days) at 23°C and a photon flux density of 30 μmol m^−2^ s^−1^. These growth conditions were optimal for observing phenotypic differences between the *m2hm3h* and WT and were also used in studies of *pif* (Leivar et al., 2012). Therefore, these growth conditions were used in all subsequent experiments. The hypocotyl length of the *m2hm3h* was significantly reduced compared to WT under both dark and light/dark conditions (Figure 2E,F,I,K). The *m2h* and *m3h* also showed shorter hypocotyl lengths than the WT, but to a lesser extent than *m2hm3h* under dark and light/dark conditions. The *m2hm3h* showed overall dwarfism and was characterized by reduced biomass compared to WT and single mutants after growth for 4 weeks (Figure 2L). These findings suggest that the underlying pathways controlling growth and development under dark or light–dark cycle conditions are impaired by mutations in *M2H* and *M3H*. They also indicate that *M2H* and *M3H* are redundantly involved in the growth of Arabidopsis.

### Phenotypes of the *m2hm3h* and *pif4pif5*

**Figure 3:**
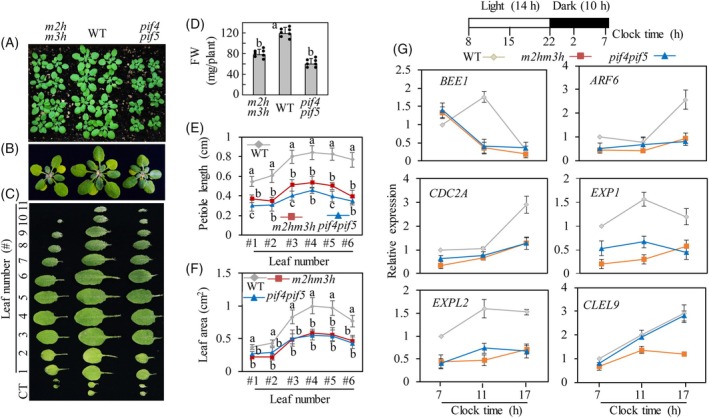
Comparison of growth phenotype between *pif4pif5* and *m2hm3h*. The plants were grown in soil under 14‐h light/10‐h dark cycles for 5 weeks (A). Representative 5‐week‐old Arabidopsis plants (B) and their detached leaves showing cotyledons (labeled CT) and true leaves (leaf number #1 to #11) (C). Quantification of fresh weight (FW; shoot biomass) (D), petiole length (E), and leaf area (F) of 5‐week‐old Arabidopsis plants. Error bars (D–F) represent mean values (*n* = 7) ± SD and individual datapoints (D) are shown as dots. (G) qRT‐PCR analysis of growth‐related genes, including *BEE1, ARF6, CDC2A, EXP1, EXPL2*, and *CLEL9*, in WT, *m2hm3h*, and *pif4pif5*. The analysis was conducted using qRT‐PCR in 5‐week‐old plants grown under a 14‐h light/10‐h dark cycle. Mean ± SD values (G) are shown for three biological replicates. Whole plants were collected at specified time points for RNA extraction followed by RNA analysis. Different letters indicate significant differences (*p* < 0.05; ANOVA, followed by Tukey's HSD post hoc tests). The white and black boxes in (G) above represent light and dark times, respectively. The numbers below the boxes represent the clock time.

Based on the dwarf phenotype of *m2hm3h* and the dark induction of *M2H* and *M3H* transcripts under the control of PIF4/PIF5 in Arabidopsis, we speculated that *m2hm3h* is phenotypically similar to *pif4pif5*. PIF4 and PIF5 promote skotomorphogenic development leading to a dwarf phenotype featuring a shorter hypocotyl and petiole in *pif4pif5*; however, *pif4* showed no clear phenotypic differences compared with WT under normal growth conditions (Lorrain et al., 2008). The morphological similarities of *pif4pif5* and *m2hm3h* plants are shown in Figure 3A–F. Compared to WT, *pif4pif5* and *m2hm3h* were overall smaller, had lower fresh weight, and had shorter petioles. Notably, the leaf areas of both *pif4pif5* and *m2hm3h* were significantly decreased compared to WT. The morphological similarity of *m2hm3h* and *pif4pif5* suggests a close regulatory relationship between *M2H/M3H* and *PIF4/PIF5* in plant‐growth signaling pathways.

### Expression of growth‐related genes in the *m2hm3h* and *pif4pif5*

The finding that *pif4pif5* and *m2hm3h* showed similar growth retardation phenotypes suggests that many genes involved in seedling growth and hypocotyl elongation are similarly regulated during the day–night cycle. These growth‐related genes are associated with the BR, auxin, and cytokinin signaling pathways, all of which are PIF‐dependent. These hormones act synergistically rather than individually, depending on the developmental stage and environmental signals (Hemerly et al., 1993; Mandava, 1988; Samalova et al., 2022). To investigate whether the expression of these growth‐related genes is synchronized in *pif4pif5* and *m2hm3h*, we investigated the diurnal changes at the transcriptional level (Figure 3G). *BEE1* (*BR‐Enhanced Expression 1*) and *CLEL9* (*CLAVATA3/EMBRYO SURROUNDING REGION‐RELATED Like 9*) are transcriptionally regulated by BR signaling and contribute to shade avoidance syndrome or root growth regulation (Cifuentes‐Esquivel et al., 2013). *BEE1* was equally downregulated in the *pif4pif5* and *m2hm3h*, whereas *CLEL9* was suppressed only in the *m2hm3h* relative to WT. Furthermore, *AUXIN RESPONSE FACTOR 6* (*ARF6*), which is induced by auxin and BR (Goda et al., 2004; Valkai et al., 2020), was equally suppressed in the *pif4pif5* and *m2hm3h*. *CDC2A* (*CELL DIVISION CYCLE 2A*) which is positively involved in cell division and proliferation and is regulated by auxin and cytokinins (Hemerly et al., 1993) was significantly suppressed in the *pif4pif5* and *m2hm3h*. In addition, *EXP1* (*EXPANSIN 1*) and EXPL (*EXP‐Like*) which function in cell expansion *via* apoplast acidification and are predicted to be direct targets of auxin, cytokinin, and BR signaling in Arabidopsis (McQueen‐Mason et al., 1992; Pacifici et al., 2018; Samalova et al., 2022) were markedly downregulated in the *pif4pif5* and *m2hm3h*. These data suggest that the dwarf symptoms observed in the *pif4pif5* or *m2hm3h* were attributable to suppression of the expression of similar growth‐related genes. Although the interactions of these genes require further investigation, the phenotype of *pif4pif5* appears to be due to the repression both the *M2H* and *M3H* genes.

### Endogenous protein levels of PIF4, DELLA, and COP1 in the *m2hm3h*

**Figure 4:**
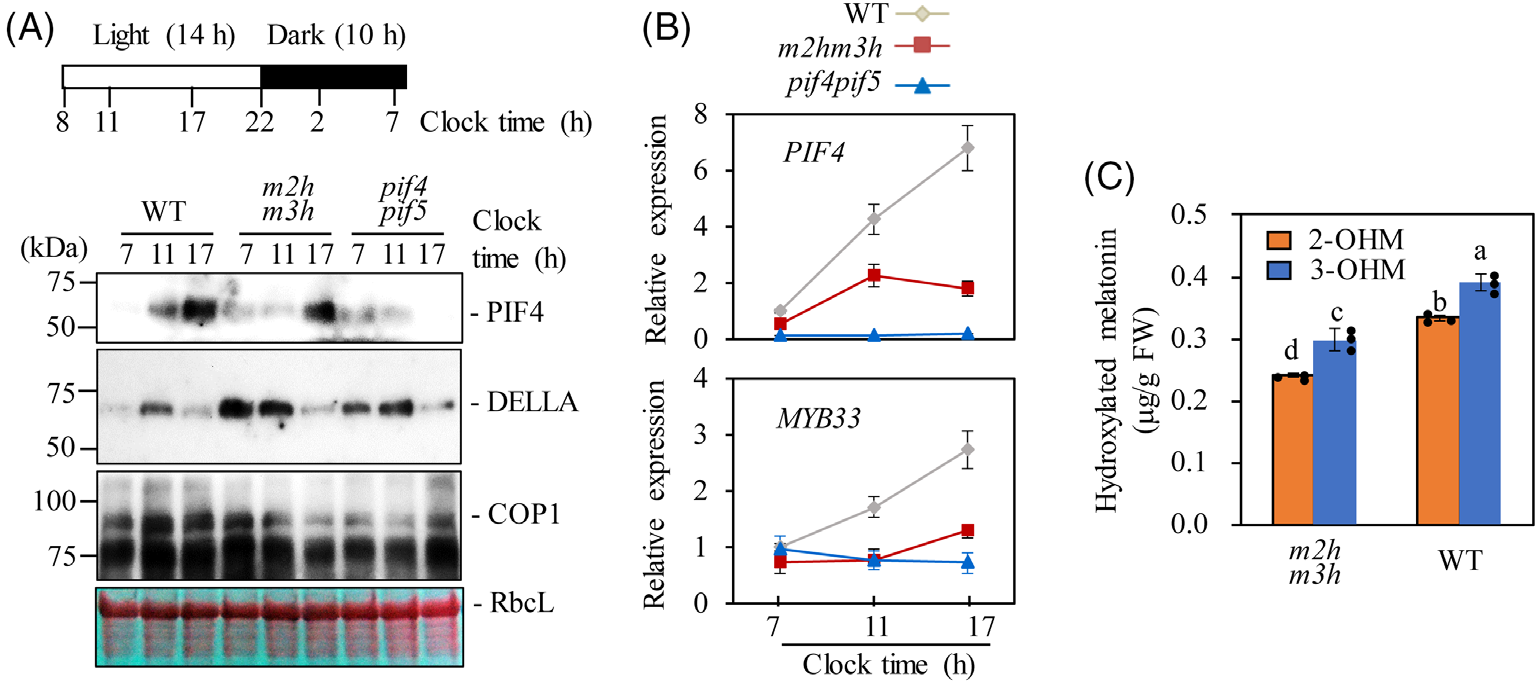
Protein and transcript levels of PIF4 and hydroxylated melatonin contents. (A) Immunoblot analysis of PIF4, COP1, and DELLA proteins. Bottom panel shows a loading control stained with Ponceau S solution showing Rubisco large subunit (RbcL) protein levels. (B) Transcript levels of *PIF4* and *MYB33* by qRT‐PCR. (C) Hydroxylated melatonin levels in WT and *m2hm3h*. Total proteins and RNA were extracted at various time points from 5‐week‐old WT, *m2hm3h*, and *pif4pif5* plants grown under 14‐h light/10‐h dark cycles at 23°C. The immunoblots were probed with specific antibodies (right). As for the quantification of 2‐OHM and 3‐OHM, 5‐week‐old leaves of *m2hm3h* and WT were abaxially infiltrated with 100 μm melatonin followed by incubation for 24 h under 14‐h light/10‐h dark cycles at 23°C. Different letters indicate significant differences (*p* < 0.05; ANOVA, followed by Tukey's HSD post hoc tests). The white and black boxes in (A) above represent light and dark times, respectively. The numbers below the boxes represent the clock time.

PIFs directly interact with phytochromes, followed by their phosphorylation and degradation (Leivar et al., 2012). Therefore, phytochromes render PIFs unstable under high red/far‐red ratio conditions, whereas PIFs accumulate in darkness. Unlike the degradation of PIF4 under high red/far‐red ratios, PIF4 protein was induced during the day under a 14‐h light/10‐h dark cycle with a photon flux density of 30 μmol m^−2^ s^−1^ in the WT (Figure 4A). However, PIF4 accumulation was markedly reduced in the *m2hm3h* compared to WT. Protein extracts from the *pif4pif5* were used to confirm the specificity of the anti‐PIF4 antibody, which served as the negative control. DELLAs (proteins containing the amino acids D‐E‐L‐L‐A at their N‐termini), particularly RGA‐like 2 (RGL2) in this report, facilitate the degradation of PIF proteins *via* the ubiquitin–proteasome system (Li et al., 2015). The nuclear protein constitutive photomorphogenic 1 (COP1), a ring‐finger E3 ubiquitin ligase, suppresses photomorphogenesis by destabilizing DELLA proteins *via* ubiquitination, thereby enhancing PIF stability and promoting plant growth (Nieto et al., 2015). In accordance with the functions of DELLAs and COP1, the DELLA protein levels were consistently higher in *m2hm3h* and *pif4pif5* compared to WT during the day/night cycles (e.g., 11 am and 5 pm) (Figure 4A). Interestingly, the elevated DELLA protein levels tended to be inversely proportional to the level of PIF4 protein in the *m2hm3h* and WT. By contrast, COP1 proteins were suppressed in the *m2hm3h* and *pif4pif5* compared to WT.

The *PIF4* mRNA level was markedly suppressed in *m2hm3h* relative to WT, suggesting that the suppression of PIF4 protein is coupled to the decreased *PIF4* transcript level (Figure 4B). GA‐responsive *MYB33* transcript expression was equally downregulated in *m2hm3h* and *pif4pif5* (Figure 4B). Accordingly, the 2‐OHM and 3‐OHM contents were significantly reduced in *m2hm3h* compared to WT, indicating that *M2H* and *M3H* are functionally coupled to the synthesis of 2‐OHM and 3‐OHM (Figure 4C). The basal levels of 2‐OHM and 3‐OHM in the WT may result from the presence of members of the *M2H* and *M3H* gene family, as in rice (Byeon & Back, 2015; Lee et al., 2016). Taken together, the level of PIF4 protein, a positive transcription factor for skotomorphogenic growth, was greatly suppressed in the *m2hm3h*, resulting in the inhibition of hypocotyl growth. The PIF4 protein suppression in *m2hm3h* was coupled to enhanced expression of DELLA proteins and the decreased COP1 protein level. These data indicate that *M2H* and *M3H*‐mediated seedling growth is associated with the regulatory mechanisms of PIF‐mediated hypocotyl elongation and the interactions with DELLA and COP1.

### Transcript expression profiles of GA‐ and BR‐biosynthetic genes in *m2hm3h* at the germination and 5‐week‐old leaf stages

In *m2hm3h* and *pif4pif5*, GA‐responsive *MYB33* transcript was markedly suppressed (Figure 4B), indicative of a decreased GA‐related gene expression. To determine whether the reduced level of *MYB33* is caused by the reduced GA‐related genes, the expression of several key GA‐biosynthetic genes—including *ent‐kaurene synthase* (*KS*), *GA20ox1*, and *GA3ox*2—was evaluated in 5‐week‐old leaves and at the germination stage. In 5‐week‐old leaves, *KS* expression in *m2hm3h* and *pif4pif5* was similar to WT, whereas *GA20ox1* expression varied between the mutants. By contrast, *GA3ox2* expression was lower in both mutants than WT during the day but higher in *m2hm3h* than WT at night. The BR‐biosynthetic gene *BR6ox2* was suppressed in both mutant plants compared to WT (Figure S1a). These results suggest that there were no differences in the expression of GA synthesis‐related genes, whereas the BR synthesis‐related gene was suppressed in *m2hm3h* and *pif4pif5* compared to WT. By contrast, in imbibed seeds at 4°C in darkness, *KS* and *GA20ox1* were downregulated at 2 days after imbibition in *m2hm3h* and *pif4pif5* compared to WT. The expression of *GA3ox2* was suppressed at 3 days after imbibition in *m2hm3h* and *pif4pif5*, suggesting suppression of GA synthesis‐related genes at the seed imbibition stage rather than the 5‐week‐old leaf stage. *BR6ox2* expression was suppressed in *m2hm3h* but not in *pif4pif5* at 2 days after imbibition (Figure S1b). Taken together, the suppression of *BEE1* (BR‐responsive gene) and *MYB33* (GA‐responsive gene) in 5‐week‐old leaves of *m2hm3h* and *pif4pif5* could be explained by the inhibition of BR‐ and GA‐synthesis‐related gene expression during the seed germination stage.

### *M2H* and *M3H* overexpression promote the growth of Arabidopsis seedlings

To examine whether overexpression of *M2H* and *M3H* leads to enhanced hypocotyl elongation, we generated *M2H* and *M3H* overexpression (M2H‐OE and M3H‐OE) Arabidopsis plants. When grown in half‐strength MS medium for 7 days, M2H‐OE and M3H‐OE exhibited longer hypocotyls than the WT; M2H‐OE had longer hypocotyls than M3H‐OE (Figure S2a,b). The expression profiles of GA‐ and BR‐biosynthetic genes were investigated in seeds imbibed at 23°C for 2 days in darkness. The expressions of *KS* and *GA20ox1* were higher in M2H‐OE than WT, whereas that of *GA3ox2* was comparable to WT (Figure S2c). In M3H‐OE, *GA3ox2* expression was elevated compared to WT. Similarly, *BR6ox2* expression was enhanced in M2H‐OE and M3H‐OE compared to WT. These results suggest that *M2H* and *M3H* have overlapping functions in promoting hypocotyl elongation by enhancing the expression of GA‐ and BR‐biosynthetic genes. The growth inhibition phenomenon of the *m2hm3h* is reversed in M2H‐OE and M3H‐OE, suggesting that these genes are directly involved in seedling growth and growth‐related gene expression.

### Effect of hydroxylated melatonin on hypocotyl elongation and gene expression profile

**Figure 5:**
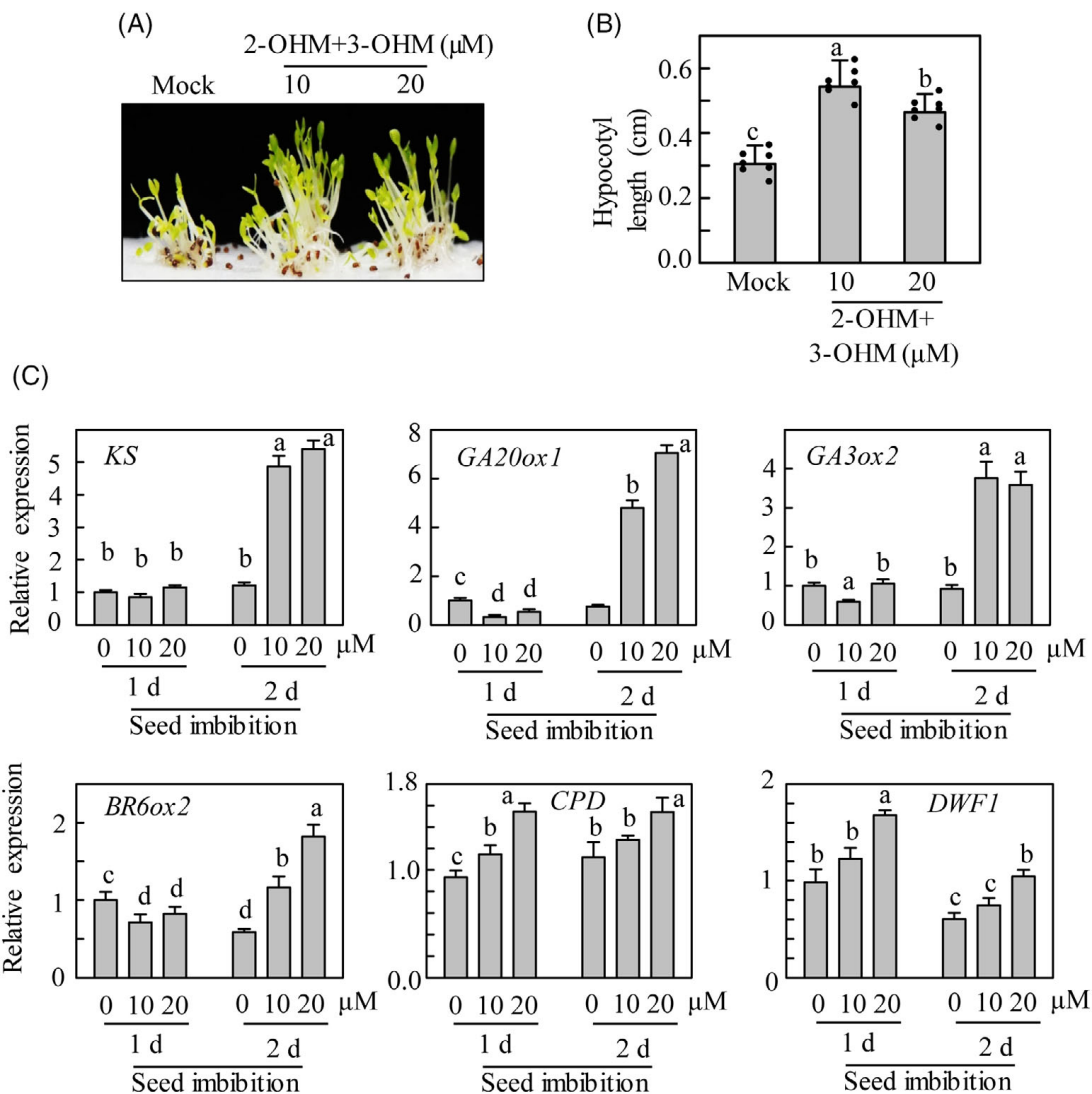
Exogenous treatment of hydroxylated melatonin and hypocotyl growth. (A) Seedling phenotypes after hydroxylated melatonin treatment (a mixture of 2‐OHM and 3‐OHM) for 7 d at 23°C under a 14‐h light/10‐h light cycle. (B) Hypocotyl length measurement. (C) Gene expression profiles by qRT‐PCR from seeds imbibed with a mixture of 2‐OHM and 3‐OHM at 23°C under dark condition. The imbibed seeds were extracted for total RNA followed by qRT‐PCR. Error bars (B) represent mean values (*n* = 7) ± SD and individual datapoints are shown as dots. Mean ± SD values (C) are shown for three biological replicates. Mock solution (1 mm MES pH 5.6 in 5 mm MgCl_2_). 2‐OHM, 2‐hydroxymelatonin; 3‐OHM, 3‐hydroxymelatonin; 2‐OHM+3‐OHM, a mixture of 2‐OHM and 3‐OHM. Different letters indicate significant differences (*P* < 0.05; ANOVA, followed by Tukey's HSD post hoc tests).

To evaluate the functions of 2‐OHM and 3‐OHM in promoting seedling growth, Arabidopsis WT seeds were exogenously challenged with a mixture of 2‐OHM and 3‐OHM for 2 days at 23°C in darkness, then rinsed and grown for 7 days. A mixture of 2‐OHM and 3‐OHM at 10 and 20 μm increased hypocotyl length by 83 and 60%, respectively, compared to mock treatment (Figure 5A,B). To determine whether hypocotyl elongation is associated with GA‐ and BR‐biosynthetic gene expression, Arabidopsis WT seeds were imbibed with a mixture of 2‐OHM and 3‐OHM at 23°C in darkness, followed by total RNA extraction for qRT‐PCR. The expression levels of the *KS*, *GA20ox1*, and *GA3ox2* were markedly enhanced 2 days after seed imbibition in seeds treated with 2‐OHM+3‐OHM but not in mock‐treated seeds (Figure 5C). No such induction was detected 1 day after seed imbibition. *BR6ox2* was also induced 2 days after seed imbibition and *CPD* and *DWF1* 1 day after imbibition. These data indicate that 2‐OHM and 3‐OHM induce the expression of GA and BR biosynthesis‐related genes at the seed imbibition stage, leading to the induction of GA and BR‐responsive genes at later leaf‐development stages in conjunction with hypocotyl elongation. Based on the fact that seed stratification induces GA synthesis at 4°C in the dark but not at 23°C, we demonstrate that 2‐OHM and 3‐OHM can replace low temperature treatment to induce GA or BR synthesis‐related genes at the seed imbibition stage.

### PIF4 binds to the E‐box motifs of the *M2H* and *M3H* promoters

**Figure 6:**
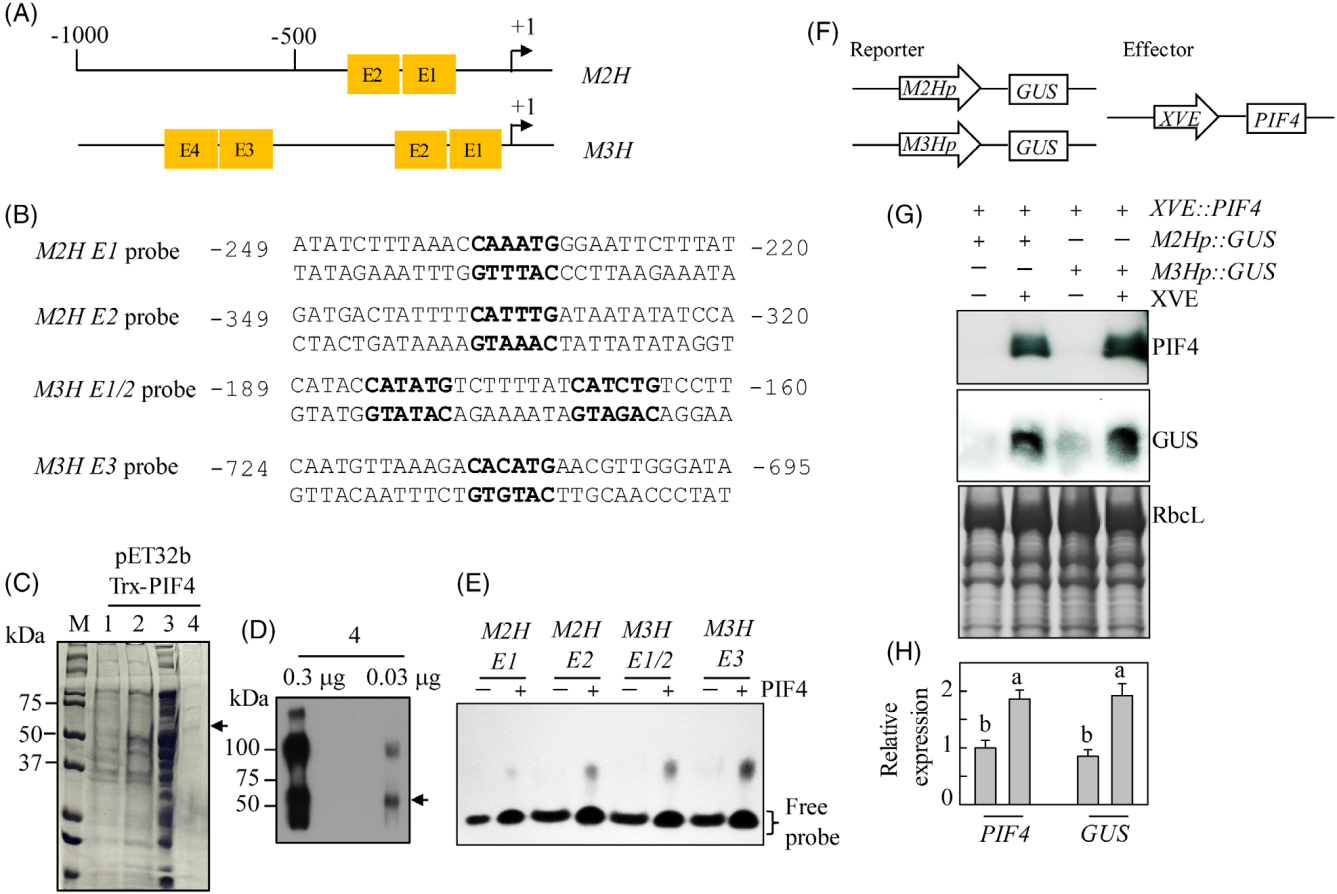
PIF4 binding to the promoter sequences of *M2H* and *M3H* and transient expression assay. (A) Schematic diagram of the *M2H* and *M3H* promoters. The positions of the E‐box are denoted. (B) The sequences of four oligonucleotides containing the E‐box elements (bold). The numbers indicate the position of the element relative to the translation start sites of *M2H* and *M3H*. (C) Affinity purification of the His‐tagged thioredoxin (Trx)‐PIF4 fusion protein. Proteins were separated by SDS‐PAGE followed by Coomassie blue staining. M, molecular size marker; lane 1, total *E. coli* protein of uninduced cells (8 × 10^6^ cells) before IPTG induction; lane 2, total *E. coli* protein of IPTG induced cells (8 × 10^6^ cells); lane 3, 20 μg of protein in supernatant after centrifugation at 10 000× ***g***; lane 4, Trx‐PIF4 protein (2.5 μg) purified by affinity (Ni‐NTA) chromatography. (D) Immunoblot analysis of the purified recombinant Trx‐PIF4 protein. The concentrations (0.3 μg and 0.03 μg) of purified recombinant Trx‐PIF4 were separated using a 10% SDS‐PAGE gel followed by Western blot analysis with an anti‐PIF4 antibody. Arrows denote the Trx‐PIF4 protein. (E) Electrophoretic mobility shift assay (EMSA) using the various E‐box nucleotides in the absence (−) or presence (+) of purified Trx‐PIF4. The position of the free probe is denoted. (F) Schematic representation of the pGWB533 vector for GUS expression under *M2H* or *M3H* promoters and the pER8 vector for XVE‐inducible PIF4 expression. *GUS*, β‐glucuronidase; *M2Hp*, promoter of *M2H*; *M3Hp*, promoter of *M3H*; *PIF4*, Arabidopsis *PIF4*; *XVE*, human estrogen receptor‐VP16. (G) Immunoblot detection of PIF4 and GUS. *Agrobacterium tumefaciens* GV2260 expressing either GUS or PIF4 were co‐infiltrated into *Nicotiana benthamiana* leaves. Two days later, leaves were treated with either XVE inducer (β‐estradiol) or mock solution, incubated for 12 h, and then collected for protein blot or RNA analysis. (H) Relative expression levels of *GUS* transcript by qRT‐PCR.

Given that the *M2H* and *M3H* transcript levels were markedly suppressed in the *pif4pif5*, but high in the *phyB*, *M2H* and *M3H* transcription is likely to be directly regulated by PIF transcription factors. Therefore, we screened for *cis*‐elements of PIFs, such as the G‐box (CACGTG) and E‐box (CANNTG), in the promoters of *M2H* and *M3H*. Although no G‐box was found in the *M2H* and *M3H* promoters, there were two E‐box motifs in *M2H* and four in *M3H* (Figure 6A,B). To investigate their possible interaction, a 30 bp DNA fragment encompassing this motif was synthesized and binding assays (EMSA; electrophoretic mobility shift assay) were conducted using recombinant PIF4 protein. The full‐length Arabidopsis gene was fused to a thioredoxin (Trx) tag using a pET32b vector and expressed in *E*. *coli*. Recombinant Trx‐PIF4 was purified and confirmed by Western blotting analysis (Figure 6C,D). Trx‐PIF4 bound to two E‐box motifs of *M2H* (*M2H* E1 and *M2H* E2) and three E‐box motifs of *M3H* (*M3H* E1/E2 and *M3H* E3). The greatest binding activity was for E3 (CACATG) of *M3H* and the weakest for the E1 (CAAATG) of *M2H*. No retardation complexes were observed in the binding mixture without PIF4 protein (Figure 6E). To confirm that PIF4 functions as a transcription factor for *M2H* and *M3H*, we generated reporter plasmids by placing the promoters of *M2H* and *M3H* upstream of the *GUS* gene using the pGWB533 vector. In addition, to transiently express *PIF4*, we created an effector plasmid by inserting *PIF4* into the pER8 vector, which is driven by the XVE‐inducible system (Byeon et al., 2014). Both plasmids were co‐infiltrated into *Nicotiana benthamiana* leaves and PIF4 expression was triggered by the addition of β‐estradiol (Figure 6F–H). The expression of the *GUS* reporter gene driven by the *M2H* and *M3H* promoters in response to the induced expression of *PIF4* was analyzed at the RNA and protein levels. PIF4 protein expression was confirmed in *N*. *benthamiana* leaves treated with β‐estradiol by protein blotting using an anti‐PIF4 antibody. PIF4 expression was accompanied by GUS protein and transcript expression. These data indicate that PIF4 functions as a transcription factor that binds to the E‐box motifs of the *M2H* and *M3H* promoters and thereby activates the transcription of *M2H* and *M3H*.

## DISCUSSION

### Distinct functions of hydroxylated melatonin in plants

Melatonin has a range of physiological functions possibly by way of its putative receptor in plants (Lee & Back, 2020; Wei et al., 2018). Melatonin affects not only growth (e.g., seed germination) but also development (e.g., senescence and flowering) and defense against abiotic stresses (e.g., cold, heat, and salt) and biotic stresses (e.g., bacteria, fungi, and viruses) (Arnao & Hernández‐Ruiz, 2019; Zhao, Chen, et al., 2019; Zhao, Yu, et al., 2019). These pleiotropic functions have been postulated to result from the combined effects of melatonin and its metabolites (e.g., 2‐OHM and 3‐OHM) (Back, 2021). Indeed, 2‐OHM rather than melatonin accelerates seed germination in Arabidopsis (Lee & Back, 2022a). In addition, melatonin induces the production of ROS in a manner mediated by 2‐OHM (Li, Wei, et al., 2018; Gao *et al*., 2019; Lee & Back, 2021). These results may be because in plants exogenous melatonin is converted into 2‐OHM and 3‐OHM by M2H and M3H, respectively (Lee et al., 2016).

2‐OHM and 3‐OHM were identified in plants by cloning of the rice *M2H* and *M3H* genes, respectively (Byeon & Back, 2015; Lee et al., 2016). Evaluating the functions of 2‐OHM in rice plants by generating transgenic rice is not satisfactory because *M2H* overexpression leads to embryogenic lethality (Choi & Back, 2019a) while *M3H* overexpression results in increased numbers of secondary tillers and panicles (Choi & Back, 2019b). By contrast, downregulation of *M2H* by RNA interference delays germination and inhibits seedling growth, whereas *M3H* suppression shows similar seedling growth to WT. To evaluate the functions of 2‐OHM and 3‐OHM in plants in detail, we used Arabidopsis for molecular, genetic, phenotypic, and physiological analyses. First, treatment of old Arabidopsis leaves with exogenous 2‐OHM, but not melatonin, in darkness promoted ROS production and senescence symptoms, suggesting that melatonin and 2‐OHM have different functions. In addition, 2‐OHM‐mediated ROS production was NADPH oxidase‐dependent, suggesting that 2‐OHM rather than melatonin promotes ROS production in plants (Lee & Back, 2021). Furthermore, 2‐OHM treatment of Arabidopsis seeds greatly promotes seed germination whereas melatonin marginally increases germination. Similarly, Arabidopsis *M2H* overexpression led to increased germination concomitant with increased ROS production, whereas the *m2h* mutation delayed germination (Lee & Back, 2022a). An Arabidopsis *m3h* exhibited reduced growth and delayed flowering in parallel with reduced expression of GA‐biosynthetic genes and *FLOWERING LOCUS T* (*FT*) (Lee & Back, 2022b). In the *snat2* in Arabidopsis, flowering is delayed because of decreased *FT* expression, but exogenous melatonin did not induce *FT* expression, whereas 3‐OHM induced *FT* gene expression, suggesting that melatonin and 3‐OHM have different functions in flowering (Lee *et al*., 2019; Lee & Back, 2022b). As our understanding of the functions of 2‐OHM and 3‐OHM in plants increases, it has become necessary to reevaluate the functions previously attributed to melatonin and to distinguish between the effects of melatonin and those of 2‐OHM/3‐OHM.

### PIF4 and PIF5‐dependent *M2H* and *M3H* expression

PIF4 and PIF5 play an important role in dark‐mediated seedling elongation. PIFs as transcriptional factors directly bind to the G‐box (CACGTG) and E‐box (CANNTG) motifs of promoters of diverse growth‐related target genes, leading to a rhythmic growth pattern, with growth rates peaking at dawn or early in the morning depending on day‐length conditions (Hornitschek et al., 2012; Martínez‐García et al., 2000; Oh et al., 2009; Shin et al., 2013). Most target genes of PIF4 and PIF5 are involved in auxin, GA, and BR biosynthesis and signaling. Thus, the overexpression of *PIF4* and *PIF5* gave rise to an elongated hypocotyl phenotype whereas their knockout mutants had a short hypocotyl phenotype. Due to their additive effects, the quadruple *pifq* and double *pif4pif5* display more severe dwarf growth patterns than the single mutants (Pham et al., 2018; Shin et al., 2009). PIF4 binds to the promoter G‐ and E‐boxes of major auxin biosynthetic genes such as *TRYPTOPHAN AMINO TRANSFERASE OF ARABIDOPSIS* (*TAA1*) and cytochrome P450 CYP79B2, indicating that its expression is closely coupled to auxin synthesis (Franklin et al., 2011). The resulting enhanced auxin synthesis induces a series of auxin‐responsive genes pivotal for auxin‐mediated seedling growth, including *EXP1* (*EXPANSIN 1*) and *EXPL* (*EXP‐Like*). *EXP1* and *EXPL* promote cell expansion *via* apoplast acidification (Xu & Zhu, 2021). Accordingly, the *m2hm3h* exhibited reduced expression of auxin‐related genes, as did the *pif4pif5* (Figure 3).

PIF4 directly activates several key BR‐biosynthetic genes (e.g., *DWF4*, *CPD*, and *BR6ox2*) (Martínez et al., 2018). The induced BR is perceived by BR receptor 1 (BRI1), followed by the dephosphorylation of brassinazole‐resistant transcription factor 1 (BZR1) and the formation of PIF4‐BZR1 heterodimer. Half of the 1500 PIF4‐regulated target genes are also regulated by PIF4‐BZR1, implicating the pivotal role of BR in the mode of action of PIF4 (Jaillais & Vert, 2012; Oh et al., 2012). In this study, the expressions of both *M2H* and *M3H* were significantly suppressed in the *pif4pif5*, indicating that their expressions depend on *PIF4* and *PIF5* (Figure 2). Indeed, PIF4 bound to the promoter E‐box of *M2H* and *M3H* (Figure 6). In addition, the finding that the expressions of both were greatly suppressed in the *bri1* strongly suggests that they are regulated by PIF4‐BZR1 heterodimer, not PIF4 alone. This hypothesis needs to be confirmed.

Similar to BR, PIF4 also enhances GA production by activating several GA biosynthetic genes such as *GA20ox1* and *GA20ox2* (Filo et al., 2015). GA is perceived by gibberellin insensitive 1 (GID1), which interacts with DELLA, a negative factor for PIF4‐mediated seedling growth (Li et al., 2015) resulting in the degradation of DELLA. Consequently, the DELLA‐mediated PIF4 regulation enables plants to modulate their growth in response to GA. The resulting degradation of DELLA induces GA‐responsive genes for seedling growth in a manner that is dependent on PIF4 (Jaillais & Vert, 2012). In this study, we found that DELLA protein levels were increased, whereas those of PIF4 protein were decreased in *m2hm3h* compared to WT, suggesting suppressed GA biosynthesis‐related genes in 5‐week‐old leaves of *m2hm3h*. However, the levels of GA biosynthetic genes in *m2hm3h* were comparable to WT, although GA‐responsive *MYB33* was downregulated in *m2hm3h*, indicative of a decreased GA‐related gene expression (Figure S1 and Figure 4).

The functions of *M2H* and *M3H* in skotomorphogenic seedling growth were evidenced by the significant increases in the *M2H* and *M3H* transcript levels in the *phyB* (Figure 2). In light, PhyB is activated and phosphorylates PIF4, which is degraded by the proteasome for photomorphogenic growth. Thus, the expressions of both *M2H* and *M3H* are associated with the skotomorphogenic development signaling module PhyB‐PIF4/5‐M2H/M3H.

The nuclear protein COP1, a ring‐finger E3 ubiquitin ligase, suppresses photomorphogenesis by destabilizing ELONGATED HYPOCOTYL 5 (HY5) and DELLA proteins through ubiquitination, thereby enhancing PIF protein stability and promoting skotomorphogenic growth in plants (Nieto et al., 2015). HY5 inhibits elongation growth by competitively binding to PIF4/5 target genes (Nieto et al., 2015). As a result, COP1 and PIF4 exhibit rhythmic protein levels typically opposite those of DELLA proteins, depending on the time of day (Li et al., 2015). The COP1 levels were reduced in the *m2hm3h* and *pif4pif5* and that of DELLA was increased, which led to the suppression of PIF4 in the *m2hm3h* (Figure 4). Therefore, the stunted seedling growth in the *m2hm3h* is attributed to the suppression of PIF4/PIF5 under the control of the COP1‐DELLA module.

### Mechanisms of 2‐OHM and 3‐OHM‐mediated skotomorphogenic growth of seedlings

In contrast to melatonin biosynthesis, which requires light (Lee et al., 2017), the production of 2‐OHM and 3‐OHM peaks in darkness in plants (Choi & Back, 2019a, 2019b). Accordingly, *M2H* and *M3H* showed daily rhythms with peak expression in darkness in Arabidopsis (Figure 2). Based on the dwarfism of the *m2hm3h* and the nocturnal expression of *M2H* and *M3H*, it is predicted that *M2H* and *M3H* regulate skotomorphogenic growth, which is characterized by the accumulation of PIFs, dephosphorylation of BZR1 (active BR pathway), and the absence of DELLA (active GA pathway) (Jaillais & Vert, 2012). As expected, the dark‐induced expressions of both *M2H* and *M3H* were greatly suppressed in the *pif4pif5* as well as the *bri1*. By contrast, their expressions were increased in the *phyB*, suggesting that they are inhibited in response to light; therefore, light‐activated PhyB triggered the degradation of PIF4. Indeed, PIF4 bound to the promoter E‐boxes of *M2H* and *M3H in vitro* and transient PIF4 expression enhanced GUS reporter expression under the control of the *M2H* and *M3H* promoters *in vivo* (Figure 6). These data suggest that *M2H* and *M3H*‐mediated seedling growth is associated with the PIF4/PIF5‐BZR‐DELLA transcription module.

Based on the PIF‐BZR‐DELLA transcription module, it is prerequisite that BR and GA biosynthetic genes are downregulated in *m2hm3h*, but not in 5‐week‐old leaves. However, GA‐ and BR‐responsive genes such as *MYB33* and *BEE1* are downregulated in *m2hm3h* in 5‐week‐old leaves, suggestive of decreased GA‐ and BR‐related gene expression (Figures 4, 5, 6 and Figure S1). These ambiguous results prompted us to examine the expression patterns of GA‐ and BR‐biosynthetic genes in an early stage of germination. As expected, the expression of all GA‐biosynthetic genes (including *KS*, *GA20ox1*, and *GA3ox2*) was suppressed in *m2hm3h* seeds imbibed at 4°C in darkness. In addition, the expression of the BR‐biosynthetic gene *BR6ox2* was also suppressed in *m2hm3h* seeds (Figure S1). Therefore, the expression of genes involved in the biosynthesis of both GA and BR was hampered at the seed germination stage in the *m2hm3h*.

To obtain direct evidence of the functions of *M2H* and *M3H* in the expression of GA‐ and BR‐biosynthetic genes, we generated *M2H* and *M3H* overexpression Arabidopsis. The expressions of GA‐ and BR‐biosynthetic genes were markedly elevated in the seed imbibition stage at 23°C in darkness (Figure S2). Finally, to confirm that *M2H* and *M3H*‐mediated 2‐OHM and 3‐OHM are responsible for the increased hypocotyl length and the induction of GA‐ and BR‐biosynthetic genes, WT Arabidopsis seeds were treated with exogenous 2‐OHM and 3‐OHM, and seedling growth and the expression of GA‐ and BR‐biosynthetic genes were analyzed. Treatment with exogenous 2‐OHM plus 3‐OHM enhanced hypocotyl length compared to mock treatment and enhanced the expression of GA‐ and BR‐biosynthetic genes at 23°C in darkness (Figure 6). These GA‐ and BR‐biosynthetic genes are induced at low temperatures (e.g., 4°C), so a mixture of 2‐OHM and 3‐OHM could replace the effect of cold treatment, as can GA (Yamauchi et al., 2004).

**Figure 7:**
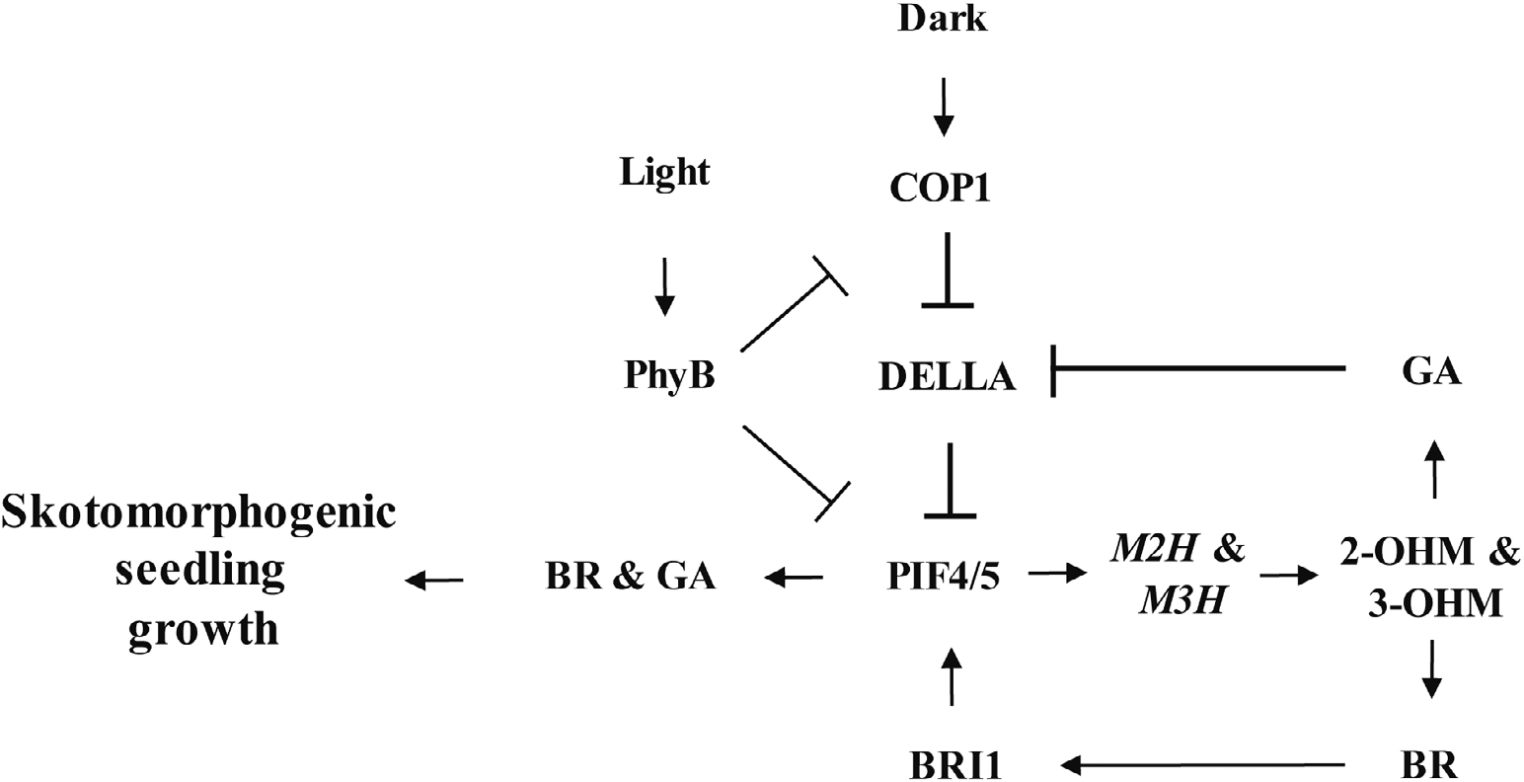
A model of hydroxylated melatonin (2‐OHM and 3‐OHM)‐mediated skotomorphogenic seedling growth *via* PIF4 and PIF5 in Arabidopsis. COP1 and PIFs play key functions in hypocotyl elongation in darkness. We found that PIF4 and PIF5 bind to the *M2H* and *M3H* promoters, and enhanced *M2H* and *M3H* transcript accumulation induces 2‐OHM and 3‐OHM synthesis, which in turn triggers the synthesis of GA and BR. GA promotes the degradation of DELLA, a negative regulator of PIF4's transcriptional activation and protein stability, which in turn induces *PIF4* mRNA and protein levels followed by a battery of GA‐ and BR‐responsive genes leading to skotomorphogenic seedling elongation. COP1 as a ring‐finger E3 ubiquitin ligase destabilizes DELLA through ubiquitination, thereby enhancing PIF4/5 protein stability. BR activates its receptor BRI1, leading to the activation of the BZR1 transcription factor, which can form PIF4‐BZR1 heterodimers to promote seedling growth, while PIF4 alone can also bind to the promoters of GA‐ and BR‐biosynthetic genes to induce the biosynthesis of these hormones. Based on the solid dependence of both PIF4/5 and BRI1, it is likely that *M2H* and *M3H* transcription is regulated by the PIF4‐BZR1 heterodimer rather than PIF4 alone. Under a light condition, PhyB is activated and inhibits both COP1 and PIF4. Collectively, the induced synthesis of GA and BR responsible for skotomorphogenic seedling growth can be achieved by 2‐OHM and 3‐OHM *via* PIF4 and PIF5. Arrows indicate positive whereas bars indicate negative regulation for skotomorphogenic seedling growth. COP1, constitutive photomorphogenic 1; 2‐OHM, 2‐hydroxymelatonin; 3‐OHM, 3‐hydroxymelatonin; GA, gibberellin; BR, brassinosteroid; BRI1, brassinosteroid receptor 1; PhyB, phytochrome B. DELLA, protein containing the amino acids D‐E‐L‐L‐A at its N‐terminal; PIF4/5, phytochrome interacting factor 4/5.

In summary, we report that hydroxylated forms of melatonin such as 2‐OHM and 3‐OHM promote the growth of Arabidopsis seedlings under the control of PIF4/PIF5 transcription factors, which bind the promoter E‐boxes of the *M2H* and *M3H* promoters. 2‐OHM and 3‐OHM are synthesized by the action of M2H and M3H, which are localized to chloroplasts and the cytoplasm, respectively. PIF4 and PIF5 are key transcription factors regulating hypocotyl seedling growth in darkness. PIF4‐dependent *M2H* and *M3H* expression suggests that PIF4/PIF5‐mediated seedling growth is linked to the expressions of both *M2H* and *M3H*. Indeed, the dwarf seedling phenotype of *pif4pif5* is similar to *m2hm3h* and the expression patterns of several growth‐related marker genes are synchronized in *pif4pif5* and *m2hm3h*. The mode of action of 2‐OHM and 3‐OHM is indicated by the downregulated expression of GA‐ and BR‐responsive genes (e.g., *MYB33* and *BEE1*) in the leaves of 5‐week‐old seedlings of *m2hm3h*, indicative of inhibition of GA and BR synthesis in the germination or early‐seedling growth stages. However, BR‐biosynthetic genes, but not GA‐biosynthetic genes, were downregulated in 5‐week‐old leaves of *m2hm3h*. By contrast, GA‐ and BR‐biosynthetic genes were markedly downregulated at the seed imbibition stage in *m2hm3h* but were significantly upregulated in M2H‐OE and M3H‐OE. Therefore, 2‐OHM and 3‐OHM induce GA and BR synthesis‐related genes in the seed imbibition stage. Indeed, the expressions of GA‐ and BR‐biosynthetic genes were considerably enhanced in WT Arabidopsis seeds exogenously challenged with a combination of 2‐OHM and 3‐OHM at 23°C in darkness. These genes are typically induced during seed stratification (4°C in darkness). Collectively, PIF4/PIF5 induce 2‐OHM and 3‐OHM synthesis, leading to GA and BR synthesis in the seed imbibition stage. Thereafter, GA triggers DELLA degradation in parallel with PIF4 induction, thereby inducing the expression of a large number of genes linked to skotomorphogenic growth of seedlings (Figure 7).

## MATERIALS AND METHODS

### Purification of AtM2H protein and enzyme kinetics

Full‐length *AtM2H* (GenBank accession no. NM115893) was cloned by RT‐PCR using the primer set (forward 5′‐GGA TCC ATG GAG GAA ACA AAT AAG‐3′; reverse 5′‐GGA TCC TCA GAG GTT TTT CTT TCT‐3′). The *AtM2H* PCR product was initially cloned to the T&A vector (RBC Bioscience, New Taipei City, Taiwan), where the *Bam*HI insert of *AtM2H* was ligated into the pGEX‐6P‐1 vector (GE Healthcare Life Sciences, Buckinghamshire, UK) to clone the AtM2H in frame with glutathione S‐transferase (GST). The *GST‐AtM2H* fusion DNA was obtained from the pGEX6P1:AtM2H plasmid using the primer set (forward 5′‐AAA AAG CAG GCT CCA TGT CCC CTA TAC TAG‐3′; reverse 5′‐AGA AAG CTG GGT TCA GAG GTT TTT CTT TCT‐3′). The resulting *GST‐AtM2H* was recombined into the pDONR221 Gateway vector (Invitrogen, Carlsbad, CA, USA) and pET300 to generate pET300‐GST‐AtM2H, which was then transformed into *Escherichia coli* BL21 (DE3) (Enzymomics, Daejeon, Korea). Expression, affinity purification, and kinetic analyses were described previously (Lee & Back, 2022b). M2H enzyme assays were carried out at pH 8.0 as described previously (Byeon & Back, 2015).

### Subcellular localization analysis of AtM2H

For the subcellular localization study of AtM2H, pER8‐mCherry was employed as described previously (Lee & Back, 2022b). The full‐length *AtM2H* cDNA was amplified by PCR using the pET300‐GST‐AtM2H plasmid as a template, using a primer set containing the *Asc*I site. The resulting *AtM2H* PCR product was digested by *Asc*I and ligated into the pER8‐mCherry vector to clone the AtM2H in frame with mCherry. The resulting pER8‐AtM2H‐mCherry plasmid was transformed into *Agrobacterium tumefaciens* strain GV2260. The leaves of four‐week‐old tobacco plants (*Nicotiana benthamiana*) were infiltrated with *Agrobacterium* strain (0.2 OD) and incubated for 2 days. Subsequently, the leaves were infiltrated with 10 μm β‐estradiol and 0.01 μm staurosporine (Sigma‐Aldrich, St. Louis, MO, USA). Treated leaves were incubated for 12 h in a humid chamber lined with wet filter paper, covered with a transparent plastic film, and incubated under growth room conditions (10 h darkness followed by 2 h dim light). Images were obtained using a Leica TCS‐SP5 confocal microscope (Leica, Wetzlar, Germany) as previously described (Lee & Back, 2022b).

### Measurement of 2‐OHM and 3‐OHM by high‐performance liquid chromatography (HPLC) analysis

Arabidopsis leaves (100 mg) were ground into powder in liquid nitrogen using the Tissuelyser II system (Qiagen, Tokyo, Japan) as described previously (Choi & Back, 2019a, 2019b). 2‐OHM and 3‐OHM were detected by HPLC using an ultraviolet (UV) detector and a fluorescence detection system (Waters, Milford, MA, USA), respectively. All measurements were carried out in triplicate.

### Plant materials and growth conditions

*Arabidopsis thaliana* (L.) Heynh. Columbia‐0 seeds (WT) were used for this study. The *m2h* (CS365315) was provided by GABI Kat (Kleinboelting et al., 2012), and *m3h* (SALK_144842) was obtained from the Arabidopsis Biological Resource Center (ABRC, Columbus, OH, USA). Genomic DNA extracted from the *m2h* and *m3h* was tested for their homozygosity by PCR screening (genotyping) using gene‐specific primers. T‐DNA insertion sites were identified in the first intron of the *M2H* gene (AT3g60290) and the second exon of the *M3H* gene (AT1g17020). The gene‐specific primers used for genotyping are as follows: For *M2H*, 5'‐GGAGGAAACAAATAAGAGTGTGG‐3' and 5'‐AAGGCCACAAATTGATCCAG‐3' and for *M3H, 5'‐ATGGAAAGCAAAAGGGGCAGC‐3' and 5'‐GATTCTCAAAGCATCTAGAT‐3'*. To obtain the *m2hm3h*, two single‐mutant plants were crossed, yielding *F*_1_ double‐heterozygous plants. Self‐pollination of the *F*_1_ plants generated an *F*_2_ population segregating both mutants. Genotyping was performed on approximately 20 *F*_2_ segregating seedlings using the PCR method. *F*_2_ seedlings homozygous for the double mutant genotypes were grown to maturity for seed collection and subsequent analyses.

To measure petiole length and leaf area, seeds (*m2h*, *m3h*, *m2hm3h*, and *pif4pif5*) were surface‐sterilized and then imbibed in the dark at 4°C for 1 day. The resulting seeds were irradiated with white light (30 μmol m^−2^ s^−1^) for 6 h and then sown in a commercial horticulture substrate consisting of a mixture of coco peat (47%), peat moss (35%), vermiculite (10%), and zeolite (7%) obtained from FarmHannong, Seoul, Korea. The substrate was prepared with a 3:1 ratio of nutrient‐deficient substrate to nutrient‐sufficient substrate. Arabidopsis was grown under a 14‐h light/10‐h dark cycle at 23°C and 60% humidity under a photon flux density of 30 μmol m^−2^ s^−1^. To measure petiole length and leaf area, true leaves were removed from 5‐week‐old plants (*n* = 7), photographed, and their values were quantified using ImageJ software (National Institutes of Health, MD, USA).

To measure the hypocotyl length of seedlings grown in the dark or light, seeds were surface‐sterilized and then imbibed in the dark at 4°C for 1 day. After irradiating the seeds with white light (30 μmol m^−2^ s^−1^) for 6 h, approximately 50 seeds were sown in rows on half‐strength MS media (2.2 g MS, 10 g sucrose, 0.5 g MES, 8 g phyto‐agar, 1 liter, pH 5.7) and grown horizontally under light/dark conditions at 23°C under a 14‐h light/10‐h dark cycle (30 μmol m^−2^ s^−1^) for 7 d or in complete darkness for 4 d. For vertical imaging, whole seedlings and the agar on which they were grown were carefully excised and placed on their sides for photography (Figure 2E,F). For gene expression analysis, 4‐ or 5‐week‐old true leaves were harvested at various time points of the light/dark cycle to extract total RNA as described below.

### Generation of *melatonin 2‐hydroxylase* ( *M2H* ) and *melatonin 3‐hydroxylase* ( *M3H* )‐overexpression *A. thaliana* plants

Full‐length *M2H* and *M3H* cDNA sequences were amplified by PCR using primers 5'‐AAA AAG CAG GCT ATG GAG GAA ACA AAT AAG‐3' and 5'‐AGA AAG CTG GGT TCA GAG GTT TTT CTT TCT‐3' for *M2H* and primers 5'‐AAA AAG CAG GCT ATG GAA GCA AAA GGG GCA‐3' and 5'‐AGA AAG CTG GGT GAT TCT CAA AGC ATC TAG AT‐3' for *M3H*. Both *M2H* and *M3H* open reading frames were subcloned into the pDONR221/Zeo vector using the BP reaction (Invitrogen). For overexpression, pDONOR 221/Zeo‐M2H or pDONOR 221/Zeo‐M3H was recombined into the plant transformation binary vector pYY63 (Lee et al., 2011) or pK2GW7 (Karimi et al., 2002), respectively *via* Gateway technology. All final constructs were transferred to *Agrobacterium tumefaciens* strain GV3101 using the freeze–thaw method and transformed into Arabidopsis Col‐0 plants using the floral dip method (Dubin et al., 2008). The second‐generation transgenic plants were molecularly verified and then used for the study. For the measurement of hypocotyl length of overexpression lines such as M2H‐OE and M3H‐OE, surface‐sterilized seeds without stratification were plated on half‐strength MS media and cultured at 23°C under a 14‐h light/10‐h dark cycle (30 μmol m^−2^ s^−1^) for 7 d (Figure S2). For gene expression analysis, seeds were imbibed in a mock solution (5.5 mm MgCl₂ and 1 mm MES, pH 5.6) at 23°C for 2 d in the dark. Seed RNA was extracted using Ribospin™ Seed/Fruit (GeneAll, Seoul, Korea).

### Protein assays

Total protein extracts were obtained from 4‐week‐old plants grown under a 14‐h light/10‐h dark cycle. Tissue samples were collected and immediately frozen in liquid nitrogen. The pulverized samples were prepared in a buffer containing 40 mm HEPES (pH 7.5), 100 mm NaCl, 1 mm EDTA, 10% glycerol, 0.2% Triton X‐100, and 1× Roche Protease Inhibitor Cocktail (Roche Applied Science, Indianapolis, IN, USA). The resuspended samples were then combined immediately with SDS sample buffer (Tris–HCl, pH 6.8, 10% SDS, 10 mm DTT, 20% glycerol, and 0.05% bromophenol blue) without centrifugation and boiling. Total protein extracts (15 μg) were separated on 14% sodium dodecyl sulfate‐polyacrylamide gel electrophoresis (SDS‐PAGE), and the amount was determined using an aliquot removed before mixing with SDS sample buffer. After electroblotting and protein transfer, the membranes were blocked in 1× TBS buffer containing 0.1% Tween 20 and 5% non‐fat milk for 1 h at room temperature. They were then incubated overnight at 4°C with anti‐PIF4, anti‐COP1, anti‐GUS, or anti‐DELLA antibodies. Secondary antibodies conjugated with horseradish peroxidase (anti‐rabbit for anti‐COP1 and anti‐DELLA, anti‐goat for anti‐PIF4) were then applied to the membranes for 1 h. Protein bands were visualized using the ECL system (Amersham Bioscience, Piscataway, NJ, USA). Primary antibodies anti‐PIF4, anti‐GUS, and anti‐DELLA, together with the secondary anti‐goat antibody, were purchased from Agrisera (Vännäs, Sweden) Additionally, anti‐rabbit secondary antibody (AP307P) was sourced from Merck Millipore (Darmstadt, Germany), and anti‐COP1 antibody was acquired from PHYTOAB (San Jose, CA, USA).

### Electrophoretic mobility shift assay

Full‐length Arabidopsis *PIF4* was amplified by PCR using a primer set (*Eco*RI containing forward primer 5′‐GAA TCC ATGGAACACCAAGGTTGG‐3′; *Xho*I containing stop codon‐less reverse primer 5′‐CTC GAG GTG GTC CAA ACG AGA ACC). The resulting PCR product was digested by *Eco*RI and *Xho*I and ligated into pET32b vector (Novagen, Merck, Darmstadt, Germany) predigested with the same restriction enzymes. pET32b‐PIF4 was expressed in *E. coli* and purified as described for AtM2H purification. Purified recombinant Trx‐PIF4 was confirmed by Western blot analysis using anti‐PIF4 as described above. A 30‐bp DNA oligonucleotide containing the E‐box of the *M2H* or *M3H* promoter was used for the EMSA assay (Bioneer, Daejeon, Republic of Korea). DNA was biotin‐labeled using the Biotin 3' End DNA Labeling Kit (Thermo Scientific, Waltham, MA, USA) and bound to purified Trx‐PIF4 protein using the LightShift® Chemiluminescent EMSA Kit (Thermo Scientific). Biotin‐labeled DNA at a final concentration of 100 fmol was incubated with the protein for 20 min at room temperature. The reaction mixtures were separated on a native 6% polyacrylamide gel in 0.5× TBE buffer. After electrophoresis, the DNA was transferred to a positively charged nylon membrane (Hybond‐N; Amersham Bioscience). Detection of DNA from the membrane was carried out according to the manufacturer's instructions (Thermo Scientific).

### Transient transcriptional expression analysis

To construct the effector vector cassette, full‐length *PIF4* cDNA was amplified by PCR using primers harboring *Asc*I sites (forward: 5′‐GGG GGC GCG CCA TGG AAA CAC CAA GGT TGG AGT‐3′; reverse: 5′‐GGG GGC GCG CCA GTG GTC CAA ACG AGA ACC GT‐3′). The PCR products were purified by gel electrophoresis and then inserted into the T&A vector (RBC Bioscience, New Taipei City, Taiwan). Next, the *Asc*I fragments of *PIF4* were ligated into the *Asc*I site of the binary vector pER8 to create the pER8‐PIF4 construct, as described previously (Byeon, Lee, et al., 2015). For the reporter vector cassettes, the promoter region of *M2H* or *M3H* was amplified by PCR using a primer set containing the *attB1* and *attB2* sites (*M2H* forward: 5′‐GGA GTT ATA TAG AGA TGC‐3′; reverse: 5′‐TGT TTT TTT GCAAAGA AAT‐3′; *M3H* forward: 5′‐ACG TAG TAA TAC CAC ACC‐3′; reverse: 5′‐TGG TAT TAA AAA CGT ATA‐3′). The gel‐purified PCR products were introduced into pDONR221/ZEO (Invitrogen) using BP Clonase II according to the manufacturer's protocol, and then transferred to the destination vector pGWB533 (non‐promoter‐GUS) (Nakagawa et al., 2007) by recombination with LR Clonase II (Invitrogen). The resulting plasmids were transformed into *Agrobacterium tumefaciens* strain GV2260 using the freeze–thaw method. Four‐week‐old tobacco leaves were then infiltrated with the *Agrobacterium* strains, which were then treated with 10 μm β‐estradiol to induce PIF4 expression.

### RNA extraction and quantitative real‐time PCR analysis

Total RNA was extracted from Arabidopsis plants using the NucleoSpin RNA Plant Kit (Macherey‐Nagel, Duren, Germany). DNaseI treatment was performed using columns (Machery‐Nagel) to remove any genomic DNA contamination. Then, 1 μg of eluted RNA was used for complementary DNA (cDNA) synthesis with the RNA to cDNA EcoDryTM Premix System (TaKaRa Biotechniques, Shiga, Japan). The resulting cDNA was diluted two‐fold, and 1 μl or 0.2 μl was used as a template for reverse transcription polymerase chain reaction (RT‐PCR) or quantitative real‐time PCR (qRT‐PCR) analysis, respectively. qRT‐PCR was performed in a Mic qPCR Cycler System (Bio Molecular Systems, Queensland, Australia) using the SYBR Green RT‐PCR Reagent Kit (Luna Universal qPCR Master Mix; NEB, Hitchin, UK) according to the manufacturer's instructions. Primer sequences for RNA analysis are provided in Table S1. The housekeeping gene *ELF1 ALPHA* (AT5g60390) was utilized for signal normalization. Gene names were attributed to the existing AGI locus code in this publication as follows: *M2H*: AT3g60290, *BEE1*: AT1g18400, *ARF6*: AT1g30330, *CDC2A*: AT3g48750, *EXP1*: AT1g69530, *EXPL2*: AT4g38400, *CLEL9*: AT5g64770, *PIF4*: AT2g43010, *MYB33*: AT5g06100, *ELF1α*: AT5g60390. All data (*n* = 3) are expressed as means ± standard deviations (SD).

### Exogenous 2‐OHM plus 3‐OHM treatment for hypocotyl growth and RNA analyses

To analyze the effect of hydroxylated melatonin treatment on hypocotyl growth, sterilized seeds (at least 50 seeds per 500 μl tube) were imbibed in mock solution containing a mixture of 2‐OHM and 3‐OHM for 2 d at 23°C. The seeds were washed once with the mock solution and then placed on two layers of moist 3 M paper (Whatman, Maidstone, UK) in a petri dish. They were incubated for 7 d in a growth room under a 14‐h light/10‐h dark cycle with a light intensity of 30 μmol m^−2^ s^−1^ before being photographed and measured for hypocotyl growth. For gene expression analysis, the seeds were imbibed in a mock solution or in a mixture of 2‐OHM and 3‐OHM (10 μm) at 23°C in the dark for 1–2 days. Concentrations of 10 and 20 μm (2‐OHM+3‐OHM) mean a mixture of 5 μm of 2‐OHM and 3‐OHM, and 10 μm of 2‐OHM and 3‐OHM, respectively. Seed RNA was extracted using Ribospin™ Seed/Fruit (GeneAll).

### Statistical analysis

All data were analyzed using the IBM SPSS Statistics 25 software (IBM Corp. Armonk, NY, USA). Means were compared using analysis of variance at a significance level of *P* < 0.05 according to Tukey's post hoc honestly significant difference test. Data are presented as means ± SD.

## AUTHOR CONTRIBUTIONS

HYL and KB designed the research, performed experiments, and wrote the manuscript.

## CONFLICT OF INTEREST

The authors declare no competing interests.

## Supporting information


**Figure S1.** Expression patterns of GA and BR biosynthesis genes. (a) Expression patterns of GA and BR biosynthesis genes in 4‐week‐old wild‐type, *m2hm3h*, and *pif4pif5* plants. White and black boxes above the panel represent periods of light and darkness, respectively. (b) Expression patterns of GA and BR biosynthesis genes in the imbibed seeds under dark at 4°C. Total RNA was from Figure 3 (a) or extracted from dark‐imbibed seeds after 1 or 2 days (b). Quantitative real‐time PCR (qRT‐PCR) was then performed for RNA analysis. Mean ± SD values are shown for three biological replicates.
**Figure S2.** Molecular and genetic characterization of *M2H* and *M3H* overexpression (M2H‐OE and M3H‐OE) Arabidopsis seedlings. (a) Seedling phenotypes of M2H‐OE and M3H‐OE Arabidopsis at 7 days after seeding at 23°C under 14‐h light/10‐h light cycles. (b) Hypocotyl length of M2H‐OE and M3H‐OE Arabidopsis. (c) Expression levels of GA‐ and BR‐biosynthetic genes in imbibed seeds for 2 d at 23°C under dark. Different letters indicate significant differences (p < 0.05; ANOVA, followed by Tukey's HSD post hoc tests).
**Table S1.** Sequences of primers used in PCR.

## Data Availability

The data that support the findings of this study are available on request from the corresponding author. The data are not publicly available due to privacy or ethical restrictions.
