# Supplementary material for: PIF4‐dependent 2‐hydroxymelatonin and 3‐hydroxymelatonin biosynthesis is involved in skotomorphogenic seedling growth in Arabidopsis thaliana
Source: Plant J. 2025 Aug 1;123(3):e70394. doi: 10.1111/tpj.70394 (PMC12316515; doi:10.1111/tpj.70394)
Supplement: Supplementary file 1 — Figure S1. Expression patterns of GA and BR biosynthesis genes. (a) Expression patterns of GA and BR biosynthesis genes in 4‐week‐old wild‐type, m2hm3h, and pif4pif5 plants. White and black boxes above the panel represent periods of light and darkness, respectively. (b) Expression patterns of GA and BR biosynthesis genes in the imbibed seeds under dark at 4°C. Total RNA was from Figure 3 (a) or extracted from dark‐imbibed seeds after 1 or 2 days (b). Quantitative real‐time PCR (qRT‐PCR) was then performed for RNA analysis. Mean ± SD values are shown for three biological replicates. Figure S2. Molecular and genetic characterization of M2H and M3H overexpression (M2H‐OE and M3H‐OE) Arabidopsis seedlings. (a) Seedling phenotypes of M2H‐OE and M3H‐OE Arabidopsis at 7 days after seeding at 23°C under 14‐h light/10‐h light cycles. (b) Hypocotyl length of M2H‐OE and M3H‐OE Arabidopsis. (c) Expression levels of GA‐ and BR‐biosynthetic genes in imbibed seeds for 2 d at 23°C under dark. Different letters indicate significant differences (p < 0.05; ANOVA, followed by Tukey's HSD post hoc tests). Table S1. Sequences of primers used in PCR. [file TPJ-123-0-s001.docx]

**Supporting Information** – Lee and Back (2025)


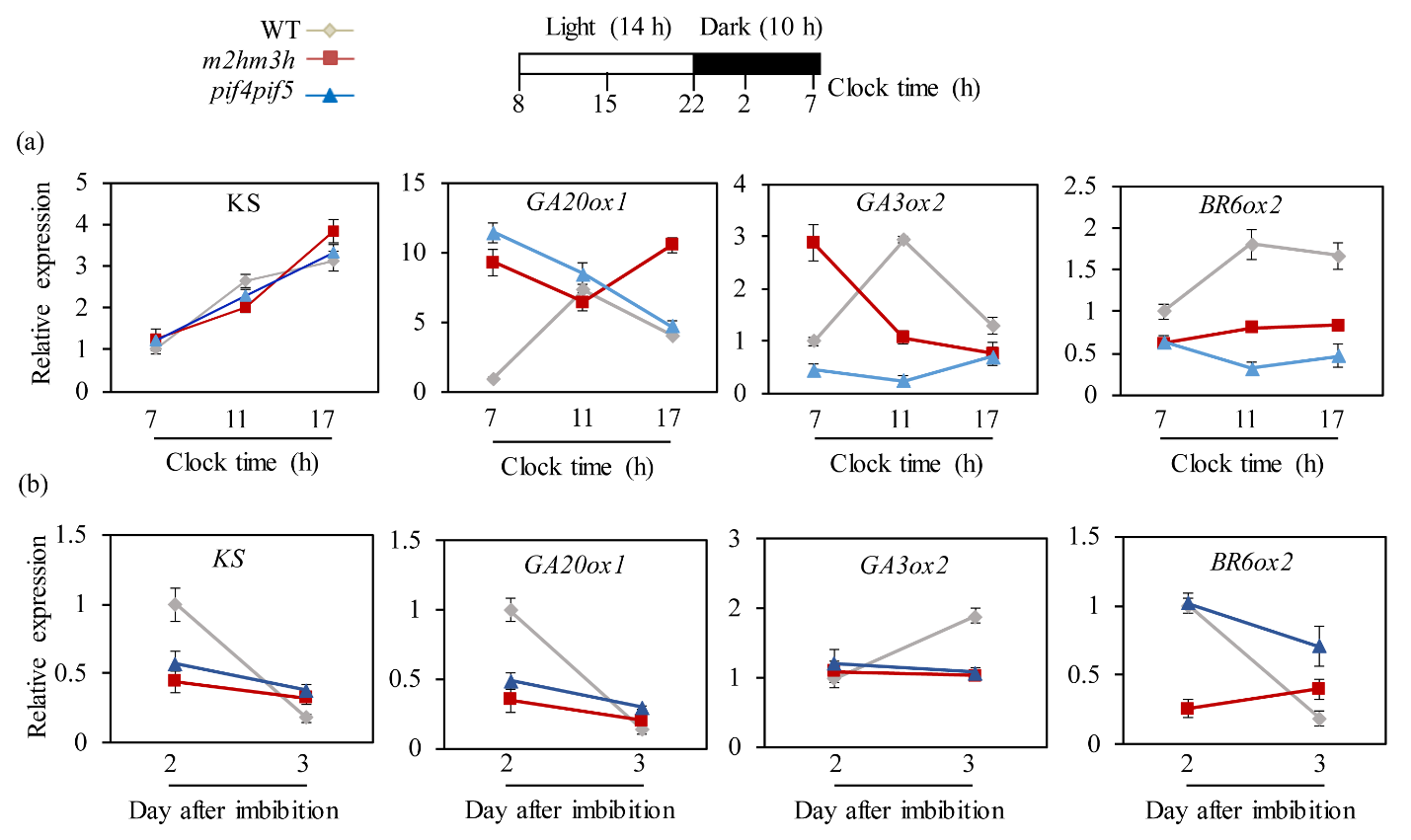


**Supplemental Figure S1** Expression patterns of GA and BR biosynthesis genes. (a) Expression patterns of GA and BR biosynthesis genes in 5-week-old wild-type, *m2hm3h*, and *pif4pif5* plants. White and black boxes above panel represent periods of light and darkness, respectively. (b) Expression patterns of GA and BR biosynthesis genes in the imbibed seeds under dark at 4^o^C. Total RNA was from Figure 3 (a) or extracted from dark-imbibed seeds after 2 or 3 days (b). Quantitative real-time PCR (qRT-PCR) was then performed for RNA analysis. Mean ± SD values are shown for three biological replicates. The white and black boxes in Figure S1 above represent light and dark times, respectively. The numbers below the boxes represent the clock time.


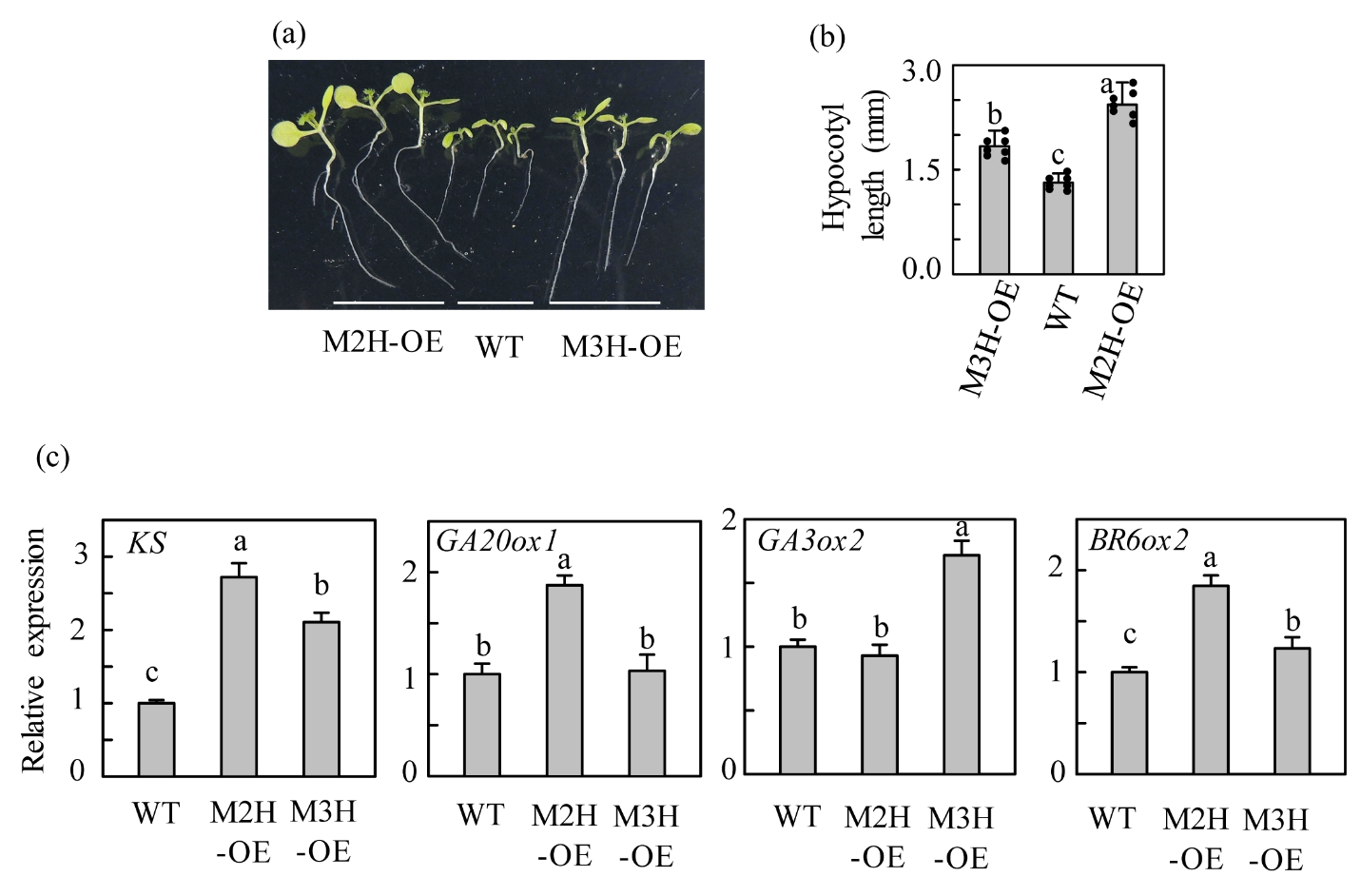


**Supplemental Figure S2** Molecular and genetic characterization of *M2H* and *M3H* overexpression (M2H-OE and M3H-OE) *Arabidopsis* seedlings. (a) Seedling phenotypes of M2H-OE and M3H-OE *Arabidopsis* at 7 d after seeding at 23^o^C under 14-h light/10-h light cycles. (b) Hypocotyl length of M2H-OE and M3H-OE *Arabidopsis*. (c) Expression levels of GA and BR biosynthetic genes in imbibed seeds for 2 d at 23^o^C under dark. Different letters indicate significant differences (p < 0.05; ANOVA, followed by Tukey’s HSD post hoc tests). Individual datapoints (n = 7) in Figure S2b are shown as dots.

**Supplemental Table S1** Primer sequences used in this study.

| Gene | Forward (5´-3´) | Reverse (5´-3´) |
| --- | --- | --- |
| M2H | GGAGGAAACAAATAAGAG TGT GG | TGC ATG CAA CTA GGT CCA AA |
| M3H | ATG GAA GCA AAA GGG GCA GC | GATTCTCAAAGCATCTAGAT |
| ELF1α | TGGTGACGCTGGTATGGTTA | CATCATTTGGCACCCTTCTT |
| BEE1 | CGAATCGCTCTTCCTTCATC | AGACGCCGCTAGTCTCTGAT |
| ARF6 | CATCAAAGGCAAAGGCAAAT | ATCAGTTGGGGGTTCATTCA |
| CDC2A | GGATCCGACCAAAAGAATCA | GGAAACGAACTCAGAAAAAGAA |
| EXP1 | GAGTGCTTGATTTTGCACGA | CGCTTCGAGAAGGGATACAA |
| EXPL2 | TTGTACCAAGGAGGCCAAAC | GGTCACCACGAACCTGAACT |
| CLEL9 | GTCGGAAAATAAGGCACGAA | CGGGATATCTTTGGATCTCAG |
| PIF4 | GCCGATGGAGATGTTGAGAT | AACGTAATGAAGTTGCACGTTT |
| MYB33 | TTGTTCTTGGAGCAACATGC | TGCATTGGCAGTTGCTAGTC |
| KS | CCAAGTTGATCTGGCAGGTA | TTGTCTCCTAAAATCAATTTTCCTC |
| GA20ox1 | AGGACCATTGGTTCCCGTAT | GTCAGGCCAGATGAATTGGT |
| GA3ox2 | CTTTGTTCCCCCATCTCCTC | TGAATTGGTTTGGGATTTGG |
| BR6ox2 | GGGAAGAGAATGGAGAAGACAA | TTAGCCCTACAAAATGACCCTTA |
| CPD | CGCTTTTCTCCTCCTCCTCT | ATCGTCGGTTCACCAAAAAG |
| DWF1 | CAAAGCCATTAGAAAGAGAAAGTGA | CAAATTTTTATATATCATTGGGCAAA |
